# Multi-environment phenotyping of ricebean (*Vigna umbellata* (Thunb.) Ohwi & Ohashi) germplasm and identification of core set for accelerating the crop improvement programs

**DOI:** 10.3389/fpls.2026.1757675

**Published:** 2026-03-20

**Authors:** Gayacharan, Dinesh C Joshi, J Aravind, D P Wankhede, Badal Singh, Prakash Kumar, S Rajkumar, Swarup K Parida, D P Semwal, Paras Sharma, Mohar Singh, Debasis Chattopadhyay, Kuldeep Singh, G. P. Singh, Amit Kumar Singh

**Affiliations:** 1 ICAR-National Bureau of Plant Genetic Resources, New Delhi, India; 2 ICAR- Vivekananda Parvatiya Krishi Anusandhan Sansthan, Almora, India; 3 ICAR- Indian Agricultural Statistics Research Institute, New Delhi, India; 4 BRIC-National Institute of Plant Genome Research, New Delhi, India; 5 ICMR‐National Institute of Nutrition (NIN), Hyderabad, Telengana, India; 6 ICAR-National Bureau of Plant Genetic Resources, R S, Shimla, India; 7 International Crops Research Institute for the Semi-Arid Tropics, Patancheru, Hyderabad, India

**Keywords:** core development, core evaluation, core hunter, diversity analysis, potential crop

## Abstract

Ricebean (*Vigna umbellata*) is a nutrient-rich rich underutilised legume crop. It is primarily grown in the uplands of India, Nepal and China. Despite its adaptation to a wide range of agroclimatic zones and resistance to various biotic and abiotic stresses, ricebean crop improvement efforts have been slow mainly because of the low levels of genetic diversity utilised in ricebean breeding. This study presents the first multi-environment phenotyping and core collection building in ricebean with 1,589 accessions maintained at the Indian National Gene Bank. The accessions were assessed in two diverse agro-ecological regions (New Delhi and Almora), indicating significant phenotypic variations for important economic traits such as days to flowering, pod length, number of seeds per pod, and seed weight. The core subsets were sampled using MStrat, PowerCore and PCSS, and CoreHunter algorithms. The sampled coresets were evaluated using diversity indices such as genetic distance, mean difference percentage (MD%), variance difference percentage (VD%), coincidence rate (CR) and variable rate of coefficient of variation. The E-EN100 approach of CoreHunter yielded the most effective representation, resulting in a final core set with 251 accessions (14.3% from the entire collection). Diversity indices, clustering methods, QQ-plots, and distributional comparisons confirmed the representativeness of the core set. Multi-environment GGE biplot analysis identified stable and high-performing accessions for early flowering, synchronous maturity, pod and seed traits, including promising genotypes such as IC351508 and IC352944 with determinate growth habit and high yield potential. The study provides a manageable subset of the entire collection, which may play a significant role in trait discovery and ricebean cultivar development.

## 1 Introduction

Ricebean [*Vigna umbellata* (Thunb.) Ohwi & Ohashi] is an annual legume grown during the rainy season. It is regarded as a minor and potential legume owing to its cultivation in limited areas, primarily as an intercrop with maize and sorghum. It is grown mainly in the hilly regions of the Indo-China region, and spreading up to Nepal and Bangladesh (Andersen, 2012). Ricebean is well-suited to multiple environmental conditions and can be grown on nutrient-deficient soils (Arora et al., 1980). It is also regarded as a marginal pulse crop that has protein and mineral-rich seeds, and tolerates several pests and diseases (Pattanayak et al., 2019). It is also used as a fodder crop in rice fallows and as green manure, and vegetable (Singh et al., 2020). Understanding the importance of the crop, in recent years, various genomic resources in ricebean have been developed, ranging from transcriptome-based marker development to reference genome assembly, and functional genomics. Initial genomic resources like gene-based SSR markers were developed in the ricebean based on the *de novo* transcriptome study (Chen et al., 2016). More recently, multiple high-quality chromosome-scale genome assemblies have been generated, giving impetus to ricebean research (Kaul et al., 2022; Guan et al., 2022; Francis et al., 2023). Genes and regulatory networks of important traits such as grain size and yield are revealed through transcriptome and genome wide association studies (Verma et al., 2022a; Ariharasutharsan et al., 2024; Sahu et al., 2024). Further, studies on variability for nutritional and antinutritional traits in ricebean germplasm have highlighted the ricebean as a potential crop in nutritional security (Sharma et al., 2023). More recently, NIR spectroscopy-based prediction models have opened the door for laying out breeding programs to improve nutritional value in ricebean (Kaur et al., 2024).

Although significant progress has been made towards genomic resources in ricebean, phenotypic characterization of genebank collections lags far behind those of similar *Vigna* group of crops such as mungbean, urdbean and cowpea. Wild and or semi-domesticated traits such as pod dehiscence, seed shattering, a determinate plant growth habit, and asynchronous flowering remain major constraints to ricebean productivity. Overcoming these limitations through focused breeding efforts is essential to enhance the competitiveness of ricebean within modern cropping systems (Pattanayak et al., 2018). In addition to these fundamental traits, enhanced grain quality, minimized anti-nutritional factors, synchronized maturity, and pod yield are critical objectives in the breeding programme (Verma et al., 2022b). To address these objectives, genetic diversity in the form of landraces, improved cultivars, genetic stocks, wild and weedy species conserved in various gene banks across the world can play a critical role (Gautam et al., 2007; Bisht and Singh, 2013; Pattanayak et al., 2019). Although over twenty high-yielding varieties of ricebean for diverse end uses like grain and fodder suited to different agro-ecological regions are developed, these were primarily developed through selection from the small-sized collections (Pattanayak et al., 2019; Singh et al., 2020; Gayacharan et al., 2024). A more efficient approach should, however, be the selection based on the multi-environment evaluations on a larger set of collections, especially for economically important traits that show a potent genotype-environment interactions. However, multi-location testing of large germplasm collections is logistically challenging. Therefore, identifying a smaller subset of accessions that captures the maximum genetic diversity present in the entire genebank collection, referred to as a core collection, as proposed by Brown (1989) is essential. Core collections become the primary genetic resources to be used by breeders or researcher in any crop improvement programs. While core collections have been developed for various *Vigna* species (Mahalakshmi et al., 2007; Ning et al., 2008; Redden et al., 2009; Schafleitner et al., 2015; Meghwal et al., 2015), no such initiatives have been undertaken for ricebean, despite its importance in agriculture of Asian countries. Keeping in view the importance of ricebean as a multipurpose pulse crop, the Indian National Genebank, New Delhi, has assembled and conserved 1760 diverse accessions of ricebean.

Therefore, this research focuses on multi-environment phenotyping of a large ricebean germplasm collection and is followed by selecting a smaller set of accessions as a core set representing the entire genetic diversity spectrum available in the ricebean germplasm maintained by the Indian National Genebank, New Delhi. This collection aims to facilitate enhanced and diversified utilization of ricebean germplasm for cultivar development, identification of trait-specific accessions, and genomic research.

## 2 Materials and methods

### 2.1 Plant material

A total of 1589 diverse ricebean germplasm collections maintained at the Indian National Genebank served as the base material to constitute the core collection. Out of 1589 accessions, 1070 are indigenous collections (ICs), and 67 are exotic collections (ECs) introduced in the genebank from various countries (**Supplementary Table 1**). The exotic collections are primarily from Nepal (24) and Japan (11) (**Supplementary Table 1**). The complete passport for the accessions utilized in the study is archived in the PGR portal of ICAR-NBPGR, New Delhi (http://pgrportal.nbpgr.ernet.in/). The Indian ricebean germplasm collections are stratified into five regions based on passport information: North Eastern Himalayan region (583 accessions), North Western Himalayan region (187 accessions), Eastern India (177 accessions), Southern India (64 accessions), and Central region (60 accessions). The largest collections are represented from Meghalaya (452 acc.), followed by Nagaland (100), West Bengal (94) and Sikkim (86). The data regarding the origin was unavailable for certain accessions, resulting in their classification under the ‘unknown’ region (452 acc.).

### 2.2 The experimental design and phenotyping

The phenotypic characterization of 1589 ricebean germplasm was conducted at two locations, viz., New Delhi and Almora. As the ricebean crop has wider adaptability, the two contrasting types of agroecology were used for its characterization. The New Delhi location is characterized by a typical semi-arid tropical climate, with coordinates of 25° 27′ N latitude and 78° 37′ E longitude, at an elevation of 275 meters above sea level. The region experiences an annual rainfall ranging from 700 to 1150 mm, with the majority occurring between July and mid-September. The mean annual maximum temperature is recorded at 32.3°C, while the mean annual minimum temperature is 17.4°C. The Almora location represents a hilly agro-ecosystem with coordinates of 29.6°N latitude and 79.7°E longitude, at an elevation of 1650 m above sea level. The region experiences around 600 to 1230 mm of rainfall and 17°C to 26°C of atmospheric temperature during the monsoon season. The experiment was conducted using an Augmented Block Design as outlined by Federer and Ragava Rao in 1975. Each accession was planted in a two-row plot of 3 meters in length, with a spacing of 45 cm between rows and 75 cm between accessions. The crop was cultivated following the standard cultural practices recommended for ricebean.

The phenotypic data was documented according to the Minimal Descriptor List developed by ICAR-NBPGR (Mahajan et al., 2000). Quantitative traits assessed were days to 50% flowering (DF), number of branches per plant (NBP), terminal leaf length (TLL) (cm), terminal leaf width (TLW) (cm), stem diameter (SD) (mm), pod length (PL) (cm), number of seeds per pod (NSP), and seed weight (SW) (g). Qualitative traits included hypocotyl colour (HYP_CLR), seedling vigour (SED_VIG), flower colour (FLR_CLR), seed colour (SED_CLR), growth habit (GRT_HBT), Plant habit (PLT_BHT), flowering behaviour (FLR_ABL), leaflet shape (LFLT_SHP), leaflet size (LFLT_SIZ), and pod colour (POD_CLR). The multi-environment evaluation of the identified core set was conducted using eight phenotypic quantitative traits, viz., TLL, TLW, DF, DM, PL, SPP, SW, and days to 80% maturity (DM).

### 2.3 Statistical analyses for sampling the core sets

The data for 8 quantitative traits were standardised to remove scale differences, as per the methodology outlined by Milligan and Cooper (1985). The adjusted mean values were estimated for all the environments in R with the package augmentedRCBD (Aravind et al., 2023). The Bartlett’s χ² test was used to evaluate the homogeneity of error variances across multi-environment datasets (**Supplementary Table 2**). The results revealed a significant environmental influence (P< 0.005) on the phenotypic expression of all quantitative traits in the ricebean germplasm across both locations. Consequently, data from a single season (2019) at New Delhi was selected for further analyses and for the development of the core set (CS). Although the two test sites differed substantially in terrain, soil characteristics, and climatic conditions, the New Delhi site exhibited superior phenotypic expression and greater phenotypic variation among genotypes. Hence, this location was found to be the most suitable for final analysis.

Quantitative data on 12 agro-morphological traits were used to identify a representative and diverse subset of accessions (core set), with a 15% threshold established for selecting the number of accessions in the diverse panel relative to the total collection. To further enhance the genetic variability and geographic representation within the core set, biased selections were implemented using the CoreHunter program. These biased selections prioritised accessions with desirable economic traits, unique or rare characteristics, and those contributing higher variance to the major principal components (PCs). Following this systematic approach, a core collection of 251 ricebean accessions was selected (**Supplementary Table 3**).

Initially, eight distinct core sets were constructed using different algorithms, namely: (a) Mstrat, (b) PowerCore, (c) PCSS (Principal Component Score Strategy), (d) average entry-to-nearest-entry distance with 100% weight (E-NE:100), (e) average accession-to-nearest-entry distance with 100% weight (A-NE:100), (f) combined E-NE and A-NE with equal weighting (E-NE:A-NE (1:1), (g) E-NE:A-NE (0.3:0.7), and (h) E-NE:A-NE (0.7:0.3). The methods (d) through (h) were implemented using CoreHunter version 3.2.1, following the genetic distance framework described by Odong et al. (2013). The Mstrat method, based on the maximization (M) strategy, explicitly increases allelic or phenotypic richness through repeated stochastic sampling (Gouesnard et al., 2001). The PowerCore algorithm applies an advanced M strategy combined with a modified heuristic search to maximize allelic richness and minimize redundancy within the core set (Kim et al., 2007). The PCSS approach utilizes principal component analysis (PCA) to eliminate collinearity among traits and identify diverse accessions according to their relative contribution (Noirot et al., 1996). The PCSS analysis was done using `rpcss` package (Aravind, 2025). The E-NE method focuses on maximizing diversity and allelic richness, whereas the A-NE method enhances the representativeness of the diversity levels in the resulting core collection (Odong et al., 2013; De Beukelaer and Davenport, 2018).

### 2.4 Comparative evaluation of core sets for designating the final core set

All eight core set types were statistically evaluated to identify the most representative subset that accurately reflects the genetic diversity present in the entire ricebean collection used in this study. Based on the parameters such as average distance between each accession and the nearest entry (A-NE), average distance between each entry and the nearest neighbouring entry (E-NE), and average genetic distances between entries (E-E) as described by Odong et al. (2013) the core-4 i.e., E-NE with 100% weightage was selected (**Table 1**). Additional diversity evaluation parameters such as mean difference percentage (MD%), variance difference percentage (VD%), coincidence rate of range (CR%), and variable rate of coefficient of variation (VR%) were also calculated for quantitative traits following the methodology of Hu et al. (2000). Consistency in mean and variance between the entire collection and the selected core set was validated using the Sign test (Basigalup et al., 1995; Tai and Miller, 2000). To further confirm the representativeness of the core set, the Mantel test (Mantel, 1967) was applied to assess the correlation between the distance matrices of the complete collection and the core subset (**Table 1**).

**Table 1:** Comparison of different core sets developed using different statistical evaluation parameters.

Parameters	Mstrat	PowerCore	PCSS	Core Hunter				
Core	Core-1	Core-2	Core 3	Core 4 E-NE:100	Core 5 A-NE:100	Core 6 E-NE:A-NE (1:1)	Core 7 E-NE:A-NE (0.3:0.7)	Core 8 E-NE:A-NE (0.7:0.3)
E-NE	0.067	0.075	0.070	0.100	0.074	0.094	0.086	0.098
A-NE	0.042	0.046	0.067	0.045	0.035	0.038	0.036	0.040
E-E	0.246	0.272	0.284	0.279	0.254	0.272	0.263	0.274
MD%	37.5	37.5	62.5	50	12.5	25	25	37.5
VD%	100	100	100	100	25	100	87.5	100
CR%	100	99.96	99.74	98.87	97.98	98.87	99.03	98.87
VR%	131.2	140.1	147.8	137.3	118.2	133.6	127.03	135.5
Class coverage %	100	100	100	100	100	100	100	100
Sign test (Mean)	1.000ns	0.480ns	0.480ns	0.480ns	0.157ns	0.480ns	0.480ns	0.480ns
Sign test (Variance)	0.005**	0.005**	0.005**	0.005**	0.005**	0.005**	0.005**	0.005**
Mantel correlation	**	**	**	**	**	**	**	**

E-NE, Average distance between each entry and the nearest neighbouring entry; A-NE, Average distance between each accession and the nearest entry; E-E, Average genetic distance between entries; MD%, Mean difference percentage; VD%, Variance difference percentage; CR%, Coincidence rate of range; VR%, Variable rate of range; PCSS, Principal component score strategy; EN100, E-NE with 100% weightage; AN100, A-NE with 100% weightage; EN50:AN50, method with equal weightage to E-NE and A-NE.

** indicate significant differences at 1% probability levels.

Once the final core set demonstrating the greatest genetic diversity and representativeness was selected, it was subjected to comparative evaluation against the entire collection for quantitative traits using the EvaluateCore package in R (Aravind et al., 2020). Statistical comparisons of means were performed using the Newman-Keuls test (Newman, 1939; Keuls, 1952) and t-tests, while the Levene’s test (Levene, 1960) was used to verify the homogeneity of variances. For comparing frequency distributions, the Wilcoxon rank-sum test (Wilcoxon, 1945) was employed as a non-parametric alternative. Visual analyses were conducted using box plots, bar charts, and area charts to explore data patterns and trends. Quantile-Quantile (Q-Q) plots (Wilk and Gnanadesikan, 1968) and additional goodness-of-fit tests, such as the Kullback-Leibler distance (Kullback and Leibler, 1951), Kolmogorov-Smirnov test (Massey, 1951), and Anderson-Darling test (Anderson and Darling, 1954) were used to visualize and assess distributional differences between the core collection and the complete dataset (**Table 2**).

**Table 2:** Comparison of summary statistics, skewness, kurtosis, and other statistical parameters between the entire collection and the derived core set for various quantitative parameters.

	Entire collection				Core set (E-NE100)				Difference between the Entire collection and the Core set E-NE100			
Trait	Range	Mean ± SE	CV (P)	IQR	Range	Mean ± SE	CV (P)	IQR	Newman-Keuls Test^a^	t-test^b^	Levene’s Test^c^	Wilcoxon Rank test^d^
DF	42.13-125.13	69.9 ± 0.37	0.21	15.00	45-120.13	71.07 ± 0.94	0.21	20.00	0.24ns	0.24ns	0.16ns	0.05ns
NBPP	2.36-14.36	3.75 ± 0.02	0.17	0.76	2.36-14.36	3.82 ± 0.06	0.26	0.92	0.18ns	0.32ns	0.00**	0.83ns
TLL	4.39-17.17	10.34 ± 0.04	0.14	1.86	4.39-17.17	10.09 ± 0.12	0.18	2.03	0.02*	0.04*	0.00**	0.07ns
TLW	2.62-11.64	7.33 ± 0.03	0.16	1.47	2.62-11.64	7.07 ± 0.09	0.21	1.62	0.00**	0.01**	0.00**	0.00**
.SD	3.52-18.94	8.93 ± 0.05	0.20	2.24	3.52-18.94	9.1 ± 0.13	0.23	2.30	0.19ns	0.24ns	0.08ns	0.28ns
PL	3.91-12.81	8.33 ± 0.03	0.12	1.29	3.91-12.04	8.33 ± 0.08	0.15	1.61	0.96ns	0.96ns	0.00**	0.98ns
NSP	3.18-12.18	7.62 ± 0.03	0.15	1.33	3.18-12.18	7.52 ± 0.09	0.18	1.41	0.21ns	0.29ns	0.00**	0.11ns
SW	1.27-22.21	5.97 ± 0.06	0.38	2.34	1.33-22.21	6.52 ± 0.2	0.50	2.95	0.00**	0.01*	0.00**	0.12ns

days to 50% flowering (DF), number of branches per plant (NBP), terminal leaf length (TLL) (cm), terminal leaf width (TLW) (cm), stem diameter (SD) (mm), pod length (PL) (cm), number of seeds per pod (NSP), and seed weight (SW) (g); SE, Standard Error; CV, Coefficient of Variation; IQR, Interquartile Range.

^a^ Newman–Keuls test is used to compare the means of the entire collection and the core set.

^b^ t-test is used to differentiate between the means of the entire collection and the core set.

^c^ Homogeneity of variance between the entire collection and the core set was tested by Levene’s test.

^d^ Differences in frequency distribution tested by the Wilcoxon rank-sum test.

^ns^indicate non-significant; * and ** indicate significant differences at 5% and 1% probability levels, respectively.

For qualitative traits, Shannon’s diversity index (H′) (Shannon, 1948) was calculated to quantify genetic diversity within both the entire collection and the core subset. Additionally, Shannon’s evenness index (J′), also known as the Shannon-Wiener evenness index, was used to evaluate the uniformity of trait states within the populations. The maximum potential diversity (H_max_) was defined as the natural logarithm of the total number of descriptor states, representing the theoretical upper limit of diversity (**Table 3**).

**Table 3:** Comparison of CC with the EC of germplasm using diversity indices for qualitative parameters.

	Shannon–Weaver index (H′)		H_max_		Evenness (J’)		Simpson’s Index (D)		McIntosh Diversity index (U)	
Traits	Entire collection	Core	Entire collection	Core	Entire collection	Core	Entire collection	Core	Entire collection	Core
HYP_CLR	0.94	1.15	1.39	1.39	0.68	0.83	0.47	0.37	0.32	0.42
SED_VIG	0.45	0.65	1.10	1.10	0.41	0.59	0.75	0.63	0.14	0.22
FLR_CLR	0.02	0.08	1.10	1.10	0.01	0.07	1.00	0.98	0.00	0.01
SED_CLR	0.29	1.98	2.30	2.30	0.13	0.86	0.32	0.18	0.45	0.62
GRT_HBT	0.36	0.61	1.10	1.10	0.33	0.56	0.83	0.67	0.09	0.20
PLT_BHT	0.21	0.24	0.69	0.69	0.31	0.34	0.90	0.88	0.06	0.07
FLR_ABL	0.64	0.65	0.69	0.69	0.92	0.94	0.55	0.54	0.26	0.29
LFLT_SHP	0.81	0.92	1.39	1.39	0.59	0.66	0.47	0.45	0.32	0.36
LFLT_SIZ	0.61	0.78	1.10	1.10	0.56	0.71	0.67	0.56	0.18	0.28
POD_CLR	0.73	0.76	1.10	1.10	0.67	0.69	0.51	0.49	0.30	0.33
Average	0.51	0.78	1.20	1.20	0.46	0.63	0.65	0.58	0.21	0.28

HYP_CLR, hypocotyl colour; SED_VIG, seedling vigour; FLR_CLR, flower ground colour; SED_CLR, seed colour; GRT_HBT, growth habit; PLT_HBT, plant habit; FLR_ABL, flowering behaviour; LFLT_SHP, leaflet shape; LFLT_SIZ, leaflet size; POD_CLR, pod colour.

**Figure 1:**
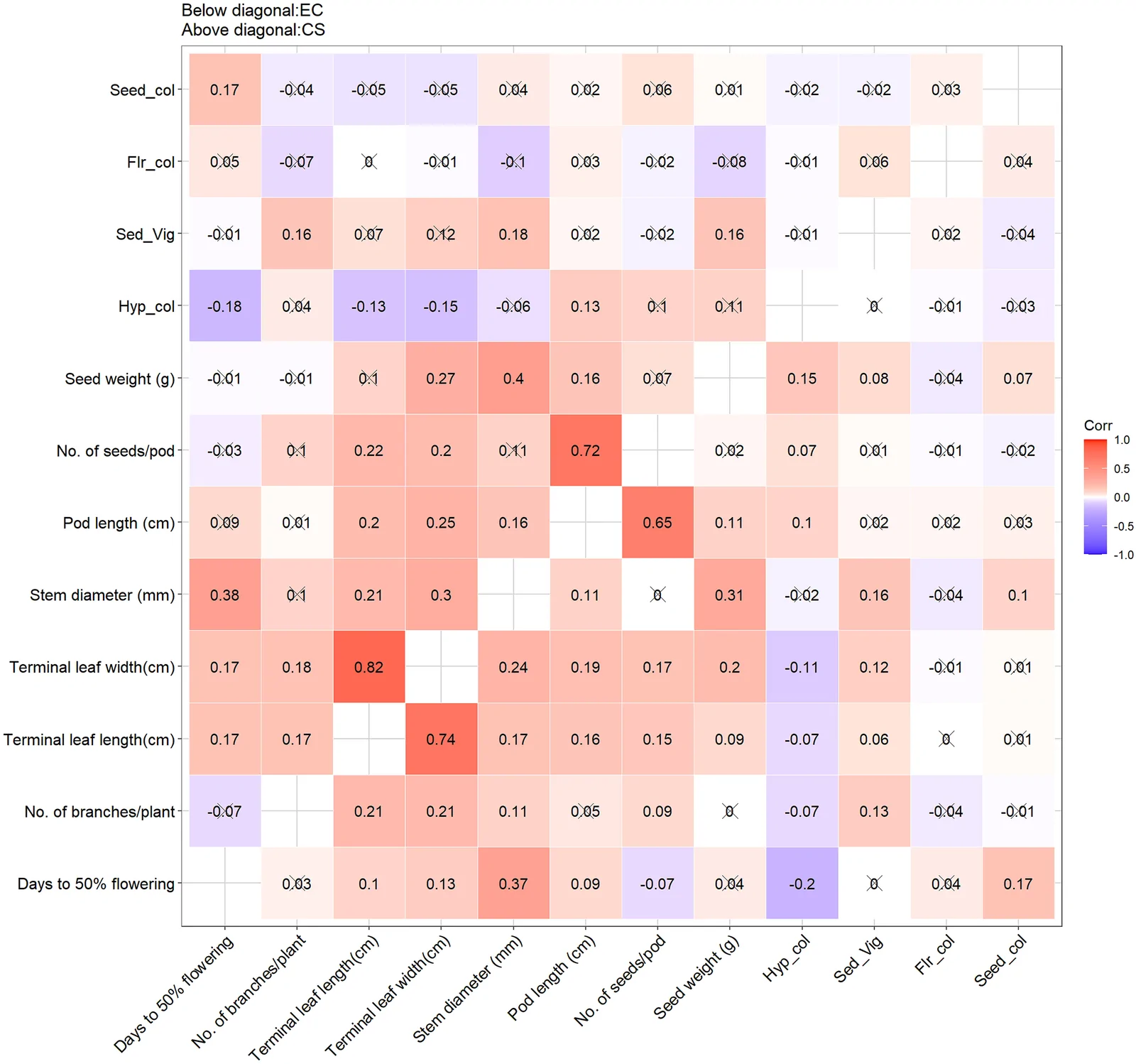
Correlation scatter matrix for quantitative traits and qualitative traits for the entire collection (EC) and core set (CS) of ricebean germplasm. The colour intensity of the grids indicates the magnitude and the direction (+/-) of the correlation. The correlation pattern indicates equitable representation of the genetic diversity in CS of the EC. (Seed_col, seed coat color; Flr_clr, flower colour; Sed_Vig, Early seedling vigour; Hyp_col, hypocotyl colour).

**Figure 2:**
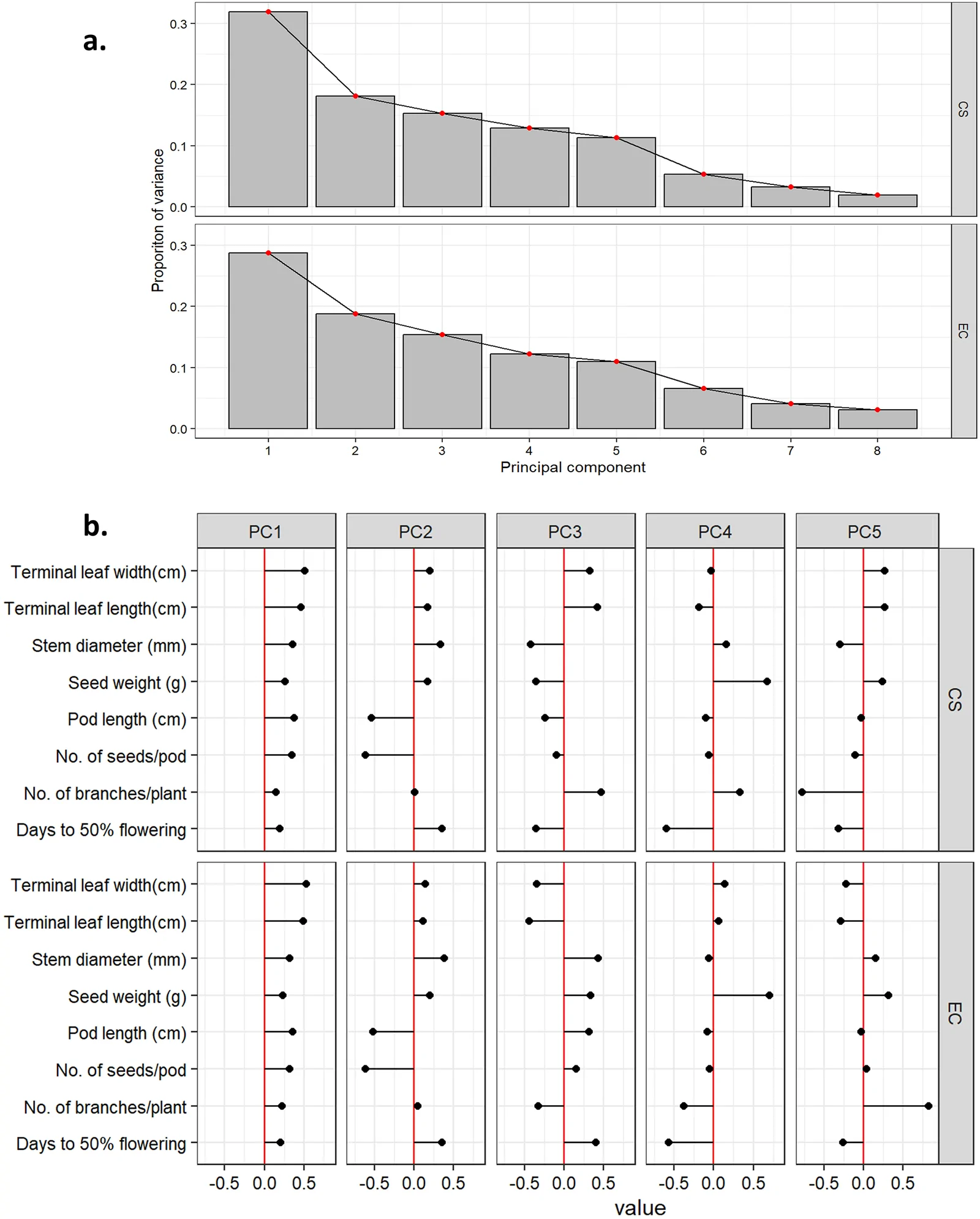
Scree plot showing gradient of variance explained by PCs in entire collection (EC) and core set (CS) **(A)** and graphical representation highlighting the relative proportion of variance for first five principal components with respect to eight quantitative traits in EC and CS **(B)**.

**Figure 3:**
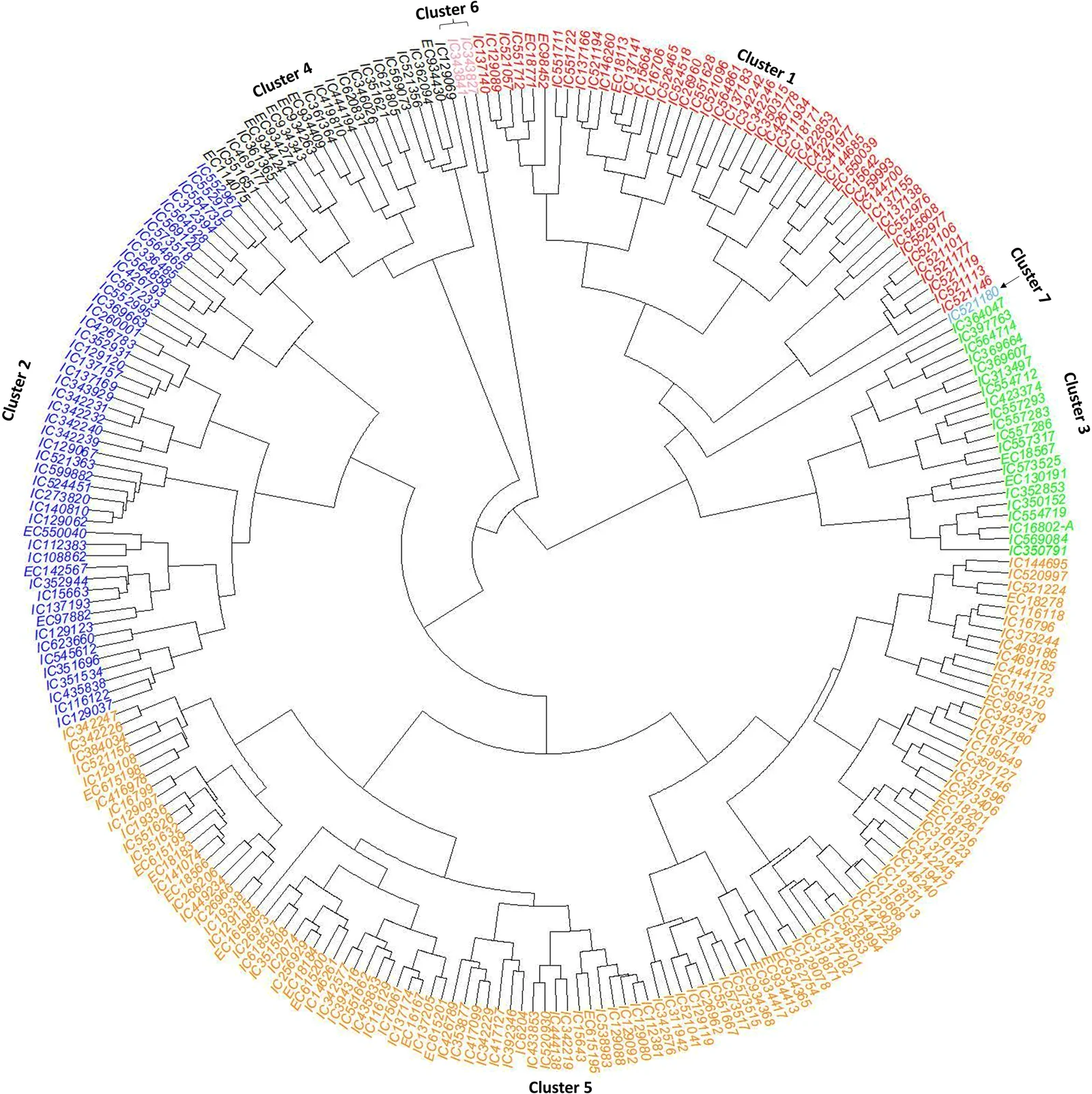
Circular agglomerative hierarchical clustering based on Ward’s minimum variance method generated using quantitative traits’ data of the core set (CS) with respective accessions at the end of each bar. The core collections were grouped into seven major clusters. The accessions’ cluster and passport information are given in the supplementary information (**Supplementary Tables S3, S4**).

To further interpret the genetic structure, Principal Component Analysis (PCA) was performed to visualize data in reduced dimensional space and to compare explained variances, directions of variation among principal components (PCs), and relationships among traits and accessions (**Table 4**; **Figure 1**). Trait interrelationships, both quantitative and qualitative, were quantified using Pearson’s correlation coefficient (Pearson, 1895), and the resulting matrix was visualized as a correlogram (Friendly, 2002) (**Figure 2**). Finally, hierarchical cluster analysis (HCA) of the core set was conducted using standardized data and Ward’s minimum variance method (Ward, 1963) to classify accessions based on genetic similarity (**Figure 3**).

**Table 4:** Principal component analysis and explained variability for the respective PCs.

	Entire Collection					Core set (Core E-NE 100)				
Parameters	PC1	PC2	PC3	PC4	PC5	PC1	PC2	PC3	PC4	PC5
Standard deviation	1.52	1.22	1.11	0.99	0.94	1.67	1.17	1.07	0.98	0.92
Eigenvalue	2.30	1.50	1.23	0.98	0.88	2.80	1.36	1.14	0.96	0.84
Proportion of Variance	28.78	18.75	15.42	12.26	11.02	35.05	17.02	14.20	11.96	10.48
Cumulative Proportion	28.78	47.52	62.94	75.20	86.22	35.05	52.07	66.28	78.23	88.71
Factors loadings										
DF	0.20	0.35	0.40	-0.57	-0.26	0.25	0.28	-0.44	-0.23	0.66
NBPP	0.23	0.05	-0.33	-0.38	0.82	0.10	0.06	0.65	0.57	0.43
TLL	0.49	0.11	-0.43	0.06	-0.29	0.46	0.02	0.37	-0.43	-0.06
TLW	0.53	0.14	-0.34	0.14	-0.22	0.49	0.10	0.29	-0.34	-0.14
SD	0.32	0.39	0.43	-0.06	0.16	0.34	0.43	-0.20	0.34	0.12
PL	0.36	-0.52	0.32	-0.08	-0.03	0.38	-0.50	-0.28	0.17	0.04
NSP	0.32	-0.62	0.16	-0.05	0.04	0.38	-0.53	-0.13	0.26	-0.04
SW	0.23	0.20	0.34	0.71	0.31	0.26	0.43	-0.17	0.34	-0.58

### 2.5 Estimation of genetic variability parameters and population structure of ricebean germplasm

Analysis of variance (ANOVA) based on the augmented block design (ABD) was performed for each quantitative trait across the entire ricebean collection using R version 4.0.4 and SAS 9.4 software (**Supplementary Table 5**). To evaluate the potential of these traits for use in crop improvement programs, key genetic variability parameters such as phenotypic variance (Vp) and genotypic variance (Vg) were estimated following the method described by Bhagasara et al. (2017). These variance estimates were subsequently used to calculate the genotypic coefficient of variation (GCV) and phenotypic coefficient of variation (PCV) as proposed by Burton (1952), along with broad-sense heritability (h²) following Lush (1940). The heritability values were classified into three categories viz., low (<30%), medium (30-60%), and high (>60%) as described by Robinson (1966). Furthermore, genetic advance (GA) and genetic gain (GG) were computed as described by Johnson et al. (1955) to assess the expected improvement under selection.

The principal component analysis (PCA) was performed on the quantitative traits using the data set on EC and CS. The optimum number of sub-populations (K) was decided using the silhouette method as described by Rousseeuw (1987). The hierarchical clustering was done using the Ward’s function, as well as the Unweighted Pair Group Method with Arithmetic Mean (UPGMA) method.

### 2.6 Multi-environment data analysis

**Figure 4:**
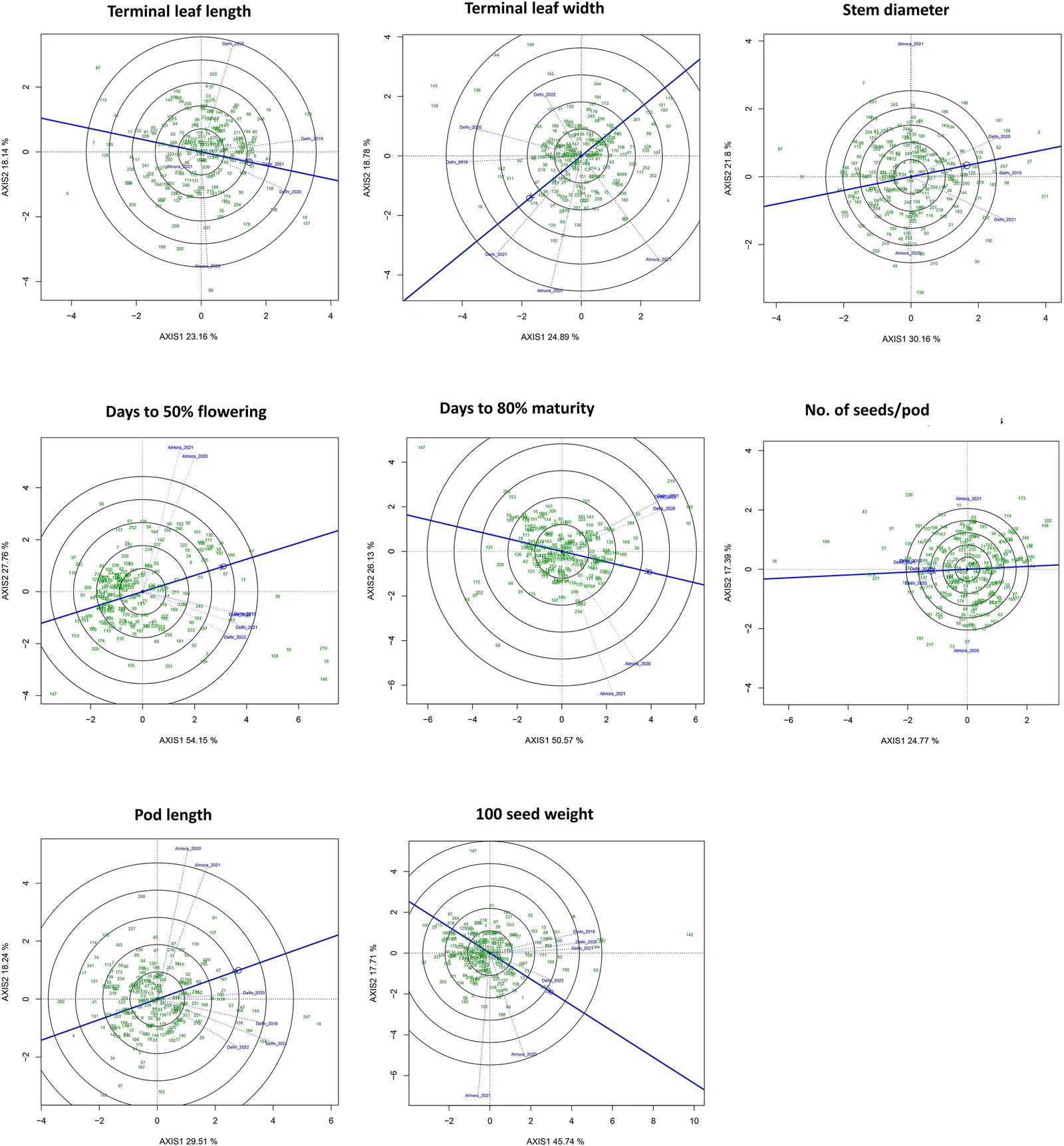
GGE biplot sowing genotype × environment (G×E) interactions of eight quantitative traits based on the first two major principal components. The blue line in each plot is the Average Environment Axis (AEA), representing the mean environment coordinate and defines the average response direction. Genotypes (green numbers in each plot) near the AEA are more stable and perform close to the average. Genotypes farther along the AEA (in the positive direction) show higher mean performance. Genotypes far from the AEA but perpendicular to it have a large G×E interaction (i.e., unstable across environments). The genotypes coded as green numbers are the serial numbers of the respective accession ID given in **Supplementary Table 3**.

To understand environmental influence on the expression of important quantitative traits *viz*., TLL, TLW, DF, DM, PL, SPP, SW, and DM, multi-environment phenotyping data on 251 accessions of core set were used, which were generated at Delhi (monsoon season of 2019, 2020, 2021 and 2022) and Almora (monsoon season of 2020 and 2021). The GGE Biplot GUI package in R employed the GGE biplot methodology, which is based on Singular Value Decomposition (SVD) of environment-centred genotype-by-environment data to simultaneously analyze genotype main effects (G) and genotype × environment interaction (GE). The model is expressed as $Y_{ij}=mu+G_{i}+E_{j}+{\left(G\times E\right)}_{ij}+\epsilon_{ij}$, where environmental means are subtracted to isolate G + GE effects. The centered data matrix is decomposed using SVD into genotype and environment scores, and the first two principal components (PC1 and PC2) are plotted to visualize genotype performance and stability, as well as environment discriminative ability and representativeness (**Figure 4**). For PCA analysis, the prcomp () function (visualized through the factoextra package) performs PCA by applying SVD to a centered and optionally scaled data matrix X=UDV′, producing orthogonal principal components that successively maximize variance. The principal component scores and loadings describe the relationships among observations (genotypes) and variables (environments), and the proportion of variance explained by each component (**Supplementary Table 8**).

## 3 Results

### 3.1 Genetic variation in ricebean germplasm collection

**Figure 5:**
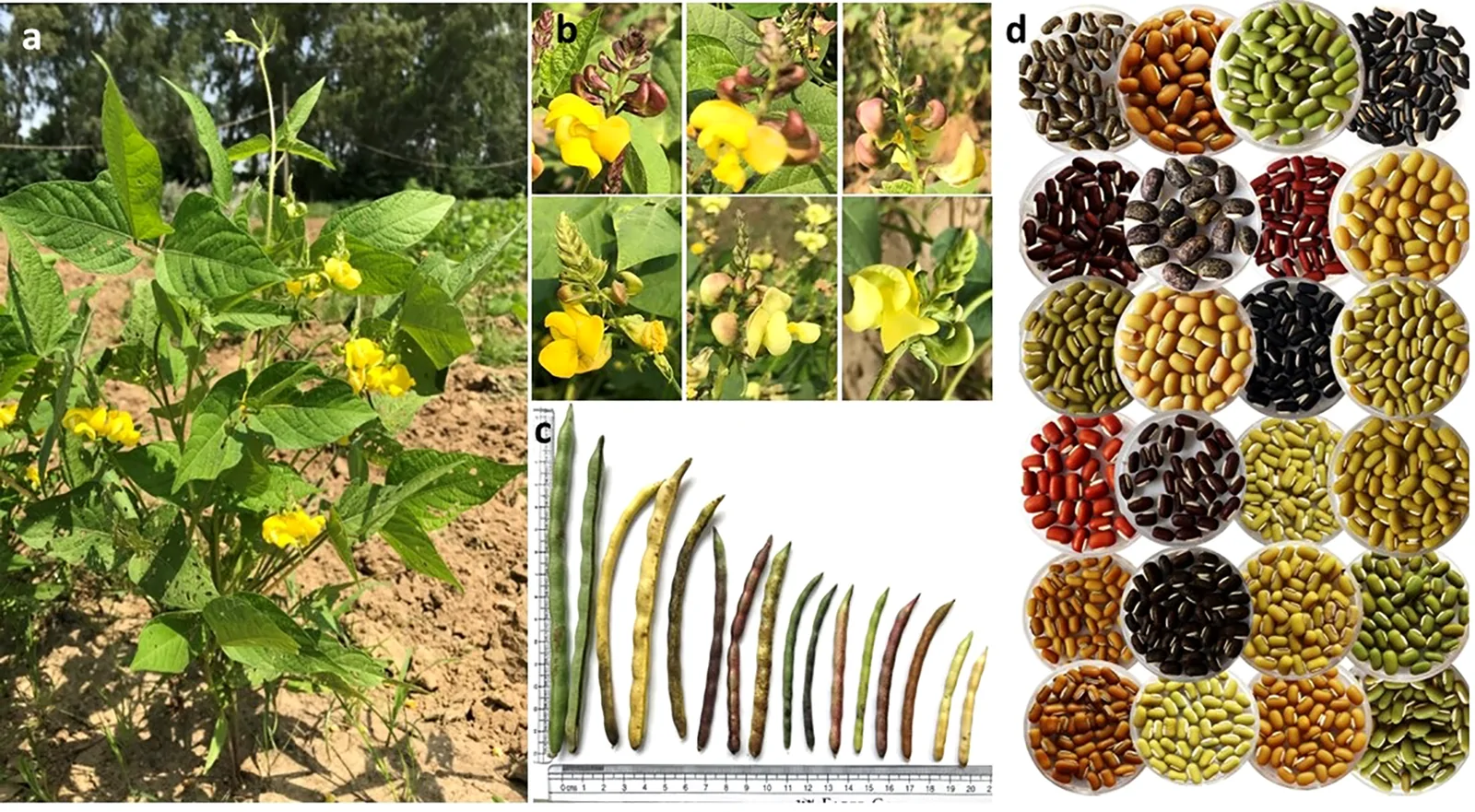
Highlights of ricebean variability. The ricebean plant typically has an indeterminate growth habit with indeterminate inflorescence (racemose), and the plant growing tip having a twining tendency also **(A)**. Ricebean germplasm has mostly yellow colour flowers, but some genotypes have slight variations **(B)**. A good amount of variation is observed for pods’ characteristics **(C)** and seed morphology **(D).**.

The ricebean germplasm exhibited substantial diversity in phenological and yield-related traits at both the locations, viz., Delhi and Almora (**Figure 5**, **Supplementary Figures S1–S6**; **Supplementary Table 8**). A wide range of variability was observed in phenological traits such as DF (42.13-125.13), NBP (2.36-14.36), TLL (4.39–17.17 cm), TLW (2.62-11.64 cm), and SD (3.52-18.94 mm). Similarly, the yield-contributing traits PL (3.91-12.81 cm), NSP (3.18-12.18), and SW (1.27-22.21 g) also showed considerable variation. Among the studied traits, SW (0.38), DF (0.21), and SD (0.20) exhibited relatively higher phenotypic variability, as indicated by their phenotypic coefficients of variation. The analysis of variance (ANOVA) on the entire collection of Delhi locations also revealed the existence of a significant amount of variation for the traits viz., PL, NSP, and SW (**Supplementary Table 5**). The broad genetic variability observed in the ricebean collection can be attributed to the inclusion of both indigenous and exotic accessions originating from diverse geographical regions represented in the Indian National Genebank. Notably, these accessions possess significant adaptive variation, having evolved across a wide range of agro-climatic zones from humid subtropical to temperate regions (**Supplementary Tables S1, S3**). The multilocation and multi-year evaluation of ricebean germplasm collection aided in the identification of trait specific genotypes that are extremely important and play a critical role in strengthening the hybridization programme to enhance the genetic base of breeding programs. Examples include genotypes with extra early flowering (42.13 days, IC351514), genotypes with very large pods (12.81 cm, IC554725), and NSP (12.18, IC521177). The collection also includes the bold-seeded accessions EC18565 (19.83 g), IC352853 (22.21 g), and IC573525 (19.71 g) compared to the check cultivars.

The North Eastern Himalayan reflected the rich diversity for the three main economic traits, namely PL, NSP, and SW, as shown by the grid mapping of indigenous ricebean accessions (**Supplementary Figures 4–S6**). To effectively use these trait-specific germplasm lines with enhanced genetic gains, their genetic parameters were estimated (**Table 5**). In ricebean, the attributes of days to 50% flowering (DF) and 100 seed weight (SW) exhibited a high degree of heritability (*h*^2^) and genetic gain (GG), indicating that the genetic diversity for these traits may be efficiently transmitted from parents to offspring. Traits including NBPP, TLW, SD, PL, and NSP exhibited a moderate amount of *h*^2^ and GG (**Table 5**; **Supplementary Figure 4**). The perusal of qualitative traits indicated that hypocotyl colour was predominantly purple (59.1%), while seed colour was mainly light green (50.8%) in the ricebean germplasm collection. The majority of ricebean germplasm has the spreading growth habit (90.7%) and indeterminate plant type (94.5%), along with asynchronous type (66.3%) flowering behaviour due to the indeterminate (racemose) type of inflorescence (**Supplementary Table 7**). Likewise, a significant proportion of the accessions demonstrated high early vigour (86.1%) and brown pod colour during near maturity (58.9%) across the entire germplasm collection. A similar pattern of diversity is observed in the core set (CS) of ricebean germplasm (**Supplementary Table 7**). The *H*′ index for seed colour and hypocotyl colour was significant and more than 1, indicating that ricebean germplasm collection exhibited a range of descriptor states for these traits (**Table 3**). In contrast, the non-significant *H*′ index (0.02) for flower colour reflected the predominance of only one descriptor state in the germplasm collection. Overall, the ricebean germplasm collection was found to be diverse for both qualitative and quantitative traits based on different diversity measures.

**Table 5:** Genetic variability parameters for quantitative traits estimated from the data on the entire ricebean collections characterized during 2019 at New Delhi location.

Traits	Mean	Vp	Vg	GCV	GCV category	PCV	PCV category	h^2^	h^2^ category	GA	GG	GG category
DF	69.90	216.65	212.38	20.85	High	21.06	High	98.03	High	29.77	42.58	High
NBPP	3.75	0.45	0.15	10.29	Medium	17.85	Medium	33.25	Medium	0.46	12.25	Medium
TLL	10.34	2.09	0.29	5.17	Low	13.99	Medium	13.66	Low	0.41	3.94	Low
TLW	7.32	1.29	0.43	8.92	Low	15.49	Medium	33.16	Medium	0.78	10.60	Medium
SD	8.92	4.05	1.65	14.40	Medium	22.56	High	40.75	Medium	1.69	18.97	Medium
PL	8.33	1.14	0.48	8.29	Low	12.84	Medium	41.69	Medium	0.92	11.05	Medium
NSP	7.62	1.22	0.48	9.05	Low	14.49	Medium	39.05	Medium	0.89	11.67	Medium
SW	5.97	5.01	4.56	35.75	High	37.47	High	91.00	High	4.20	70.35	High

Vp, phenotypic variance; Vg, genotypic variance; PCV, phenotypic coefficient of variation; GCV, genotypic coefficient of variation; h^2^, broad sense heritability; GA, genetic advance; GG, Genetic gain.

### 3.2 Extraction and composition of ricebean core

The ricebean core was assembled using four programs: CoreHunter, Mstrat, Power Core, and Principal Component Score Strategy (PCSS) using phenotypic data on 12 morpho-agronomic traits. Each of the four employed programs demonstrated the ability to extract a varied assortment of individuals, exhibiting a wide range of retention and coefficient of variation as described in previous studies (Odong et al., 2013; Krishnan et al., 2014; Archak et al., 2016). A comparison of average distances, specifically E-NE, A-NE, and E-E, across the core sampling methods indicated that the core set (CS) was effectively extracted from the entire collection (EC), demonstrating a high retention of genetic diversity (**Table 1**). The class coverage and the phenotype retention were 100% for all types of the core sets extracted. However, the variation was observed for other statistical parameters (**Table 1**). For example, genetic distances as described by Odong et al. (2013), MD%, VD%, CR%, and VR% as described by Hu et al. (2000) showed differences among core extraction methods used in this study (**Table 1**). The MD %, VD %, CR% and VR % are the standard parameters for estimating the quality and robustness of core collections (Zhang et al., 2012). Hu et al. (2000) assert that core collections with a CR % above 80% and a VR % surpassing 100% are deemed the most representative of the genetic diversity within the base collection. The VR for the eight quantitative traits was greater than 100% in the ricebean core collection, yielding an average variable rate of 125.93% across all traits. The higher CR (97.69%) demonstrates the extent to which the variability of quantitative traits has been accurately represented in the core collection derived from the entire collection. Among the strategies, E-NE with 100% weightage (E-NE:100) method of Core Hunter program, was found relatively superior in terms of diversity maximization (E-NE = 0.1), better representation (A-NE = 0.045), VD% (100) and VR% (137.3) (**Table 1**).

As expected, a bias towards Indian germplasm (IC) in the core set was observed, as most of the collections used in this study are of Indian origin (**Supplementary Table 1**). The distribution by source revealed that 85.26% of accessions of the core set were sampled from India, 14.74% from other countries, particularly from Asia. Among the Indian germplasm accessions in the core set, the maximum accessions (32.67%) originated from the North-eastern region followed by the North-western region (12.35%) (**Supplementary Table 1**).

### 3.3 Evaluation of the core sets and assessment of the selected core set

**Figure 6:**
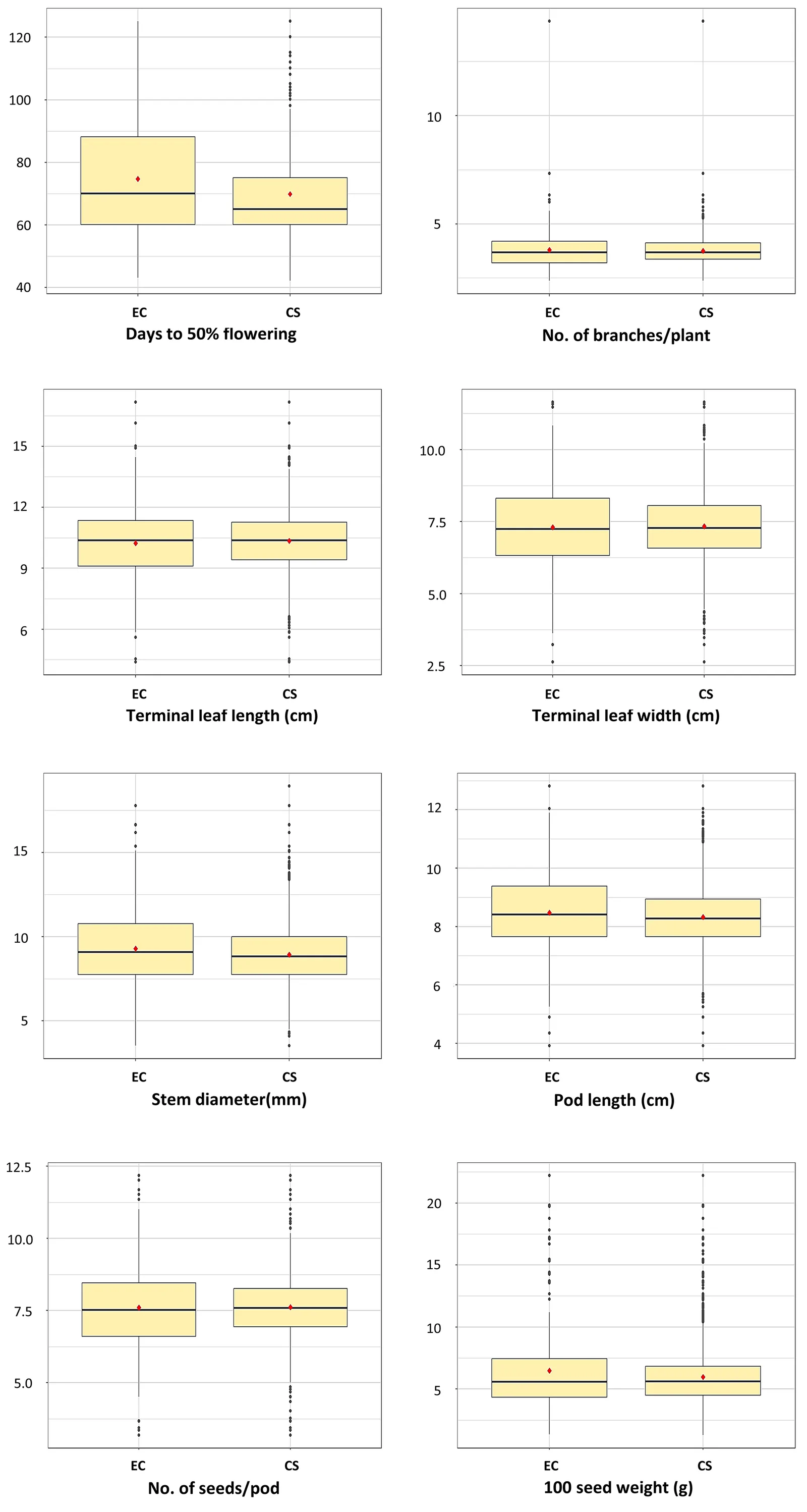
Frequency distribution of through boxplots highlighting the distribution variability of eight quantitative traits across the entire collections (EC) and the core set (CS). The figure also shows the comparison of the variability distribution between EC and CS.

**Figure 7:**
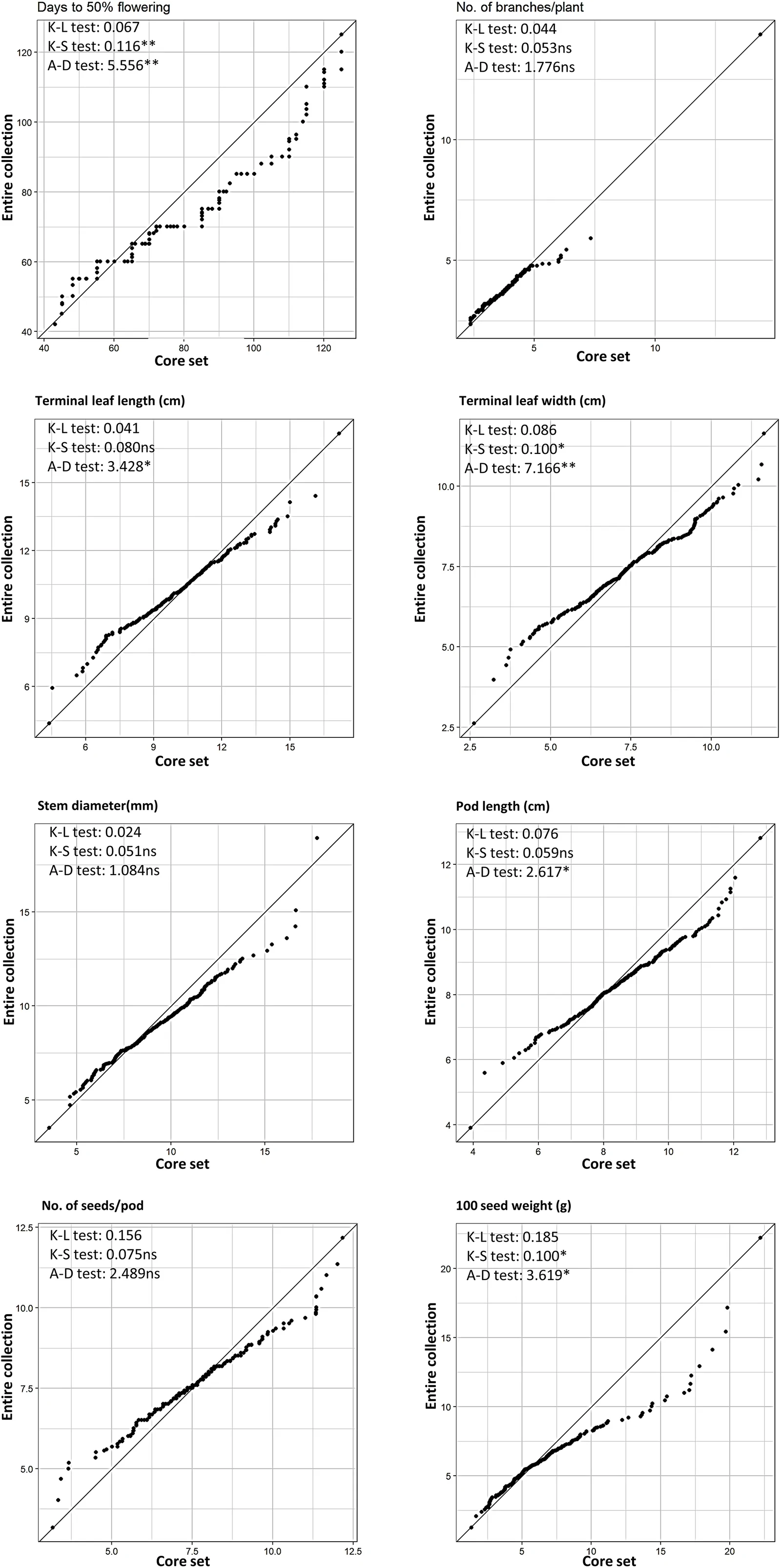
Quantile-Quantile (QQ) plot along with the Kullback-Leibler distance (K-L), Kolmogorov-Smirnov (K-S), and Anderson-Darling **(A–D)** goodness of fit test for 8 quantitative data sets of the entire collections (EC) and core set (CS).

All descriptor states (trait variants) for four qualitative traits in the entire collection were represented in the core set, suggesting that the core set likely encapsulated the allelic diversity of the entire collection (**Supplementary Table 7**). Homogenous distribution of phenotypic classes of qualitative traits in the entire and core collection indicated that the sampling technique to constitute the core collection was appropriate, and the total diversity of qualitative traits present in the entire collection is represented in the core collection. Similarly, all the geographical regions of India are covered in the core set represented, indicating a good representation of niche diversity in the core collection (**Supplementary Tables S1, S2**). Other statistical parameters, such as the range of variation, mean, and interquartile range (IQR) of distribution, also showed a good representation of diversity magnitude and its pattern across the quantitative traits (**Table 2**; **Figure 6**). In contrast to traditional comparison of means or variances, the QQ plot provides a superior visual representation of the differences in distribution of a specific trait between the core collection and the base collection (Odong et al., 2013). The QQ plots for eight quantitative traits (**Figure 7**) indicate that the distribution of the entire ricebean collection is optimally represented by the established core collection of 251 accessions.

Shannon’s diversity index (*H’*) serves as a metric for assessing allelic richness and evenness in diversity studies. A low value of *H’* signifies a highly unbalanced frequency distribution for a specific trait and indicates a narrow genetic base. The computed *H’* indices for the qualitative traits exhibited a consistent pattern of diversity across both the entire and core collections, with no significant differences detected for any of the traits (**Table 3**). The mean *H’* indices for all traits in the selected core set (CS) are similar to those of the entire collection (EC).

### 3.4 Correlation, principal component analysis (PCA) and hierarchical clustering of the entire set *vs* the core set

The silhouette method was employed to identify the optimum number of sub-clusters (k) on ricebean core set (CS). The highest average silhouette score was 7, which is considered optimum for the CS to group the ricebean collections in distinct groups (**Supplementary Figure 10**). Based on this information, the 251 accessions of ricebean CS were sub-grouped using Ward’s function (**Figure 3**) and the UPGMA method (**Supplementary Figure 12**). Although both methods grouped the accessions into seven sub-clusters, a noticeable difference is observed for the number of accessions in clusters (**Supplementary Table 4**). The correlation patterns among traits were almost similar for both populations i.e., entire collections (EC) and core set (CS) (**Figure 1**). Significant correlations are observed between terminal leaf length and Terminal leaf width, number of seeds/pod and pod length, seed weight and seed diameter, and days to 50% flowering and stem diameter (**Figure 1**). The principal component analysis revealed that 78% of the overall variance in the core dataset could be captured by the first four PCs. This value was comparable to that of the entire ricebean collection (75%), indicating the degree to which the core set preserved genetic variation. Factor loadings for the quantitative traits revealed that PC1 explained variation in terminal leaf length and width and PC2 in pod length and number of seeds per pod for both entire and core collection in ricebean germplasm (**Table 4**). In PC3, the number of branches per plant was the primary contributor to the core set, but in the base collection, stem diameter had the highest contribution. Similarly, for PC4, seed weight was the predominant contributor in the entire collection, while the number of branches per plant had the highest impact in the core set.

The hierarchical clustering further revealed the relationships among the accessions with respect to the quantitative phenotypic data (**Figure 3**). Although the trait’s grid mapping (**Supplementary Figures S1-S3**) showed traits’ diversity relationship with the geographical location, hierarchical clustering based on the eight quantitative traits showed that trait variation has no relation to the geographical origin of the ricebean collections.

### 3.5 Multi-environment analysis and identification of trait-specific accessions in the core collection

The graphical representation of GGE (Genotype + G×E) biplot analysis revealed genotype performance relative to environments (the “which-won-where” pattern) and on environment discrimination/representativeness (**Figure 4**). The traits such as DF, DM and SW recorded the highest amount of variation within the first two PCs. The DF, DM and SW captured 82%, 77% and 63% of variation, respectively (**Figure 4**; **Supplementary Table 8**). The analysis indicates that trait specific response of genotype (G) and G×E. Further PCA analysis of multi-environment data of core set accessions indicates a suitable environment for the trait expressions (**Supplementary Table 8**). GGE analysis for DF revealed 54.15% variation in axis 1 (PC1) and 27.76% variation in axis 2 (PC2). Accessions such as IC520997, EC18261, IC129123, IC137174, IC16796 and IC361364 are medium flowering duration and showed stable expression across the environments. Accessions such as IC351508, IC520997, IC129123, EC18261, IC137174, IC16796 and IC361364 showed consistent early flowering behaviour. Similarly, for the DM trait, the accessions such as EC18261, EC934368, IC351508, and IC469177 showed early maturity (**Figure 4** and **Table 6**). The late maturing (long-duration) accessions such as IC545612, IC545608, IC552967, IC552976 and IC552995 with high-biomass are suitable for regions with long post-monsoon growing periods. Ricebean accessions IC545608, IC422927 and IC449234 in the Delhi environment showed higher pod length, whereas others, such as IC569084, IC557317, IC352853, IC422927, IC422853 and IC449234 showed higher seed weight (HSW) in the Delhi environment (**Figure 4**; **Table 6**). As seed weight is the critical trait in crop yield improvement breeding, these accessions can serve as promising donors for ricebean crop improvement programs. Overall, across traits, PC1 explained 23-54% and PC2 explained 17-28% of the total G + GE variation, confirming moderate to strong G × E interactions. The analysis also revealed that Delhi location was moderately to highly discriminative for traits such as HSW, SD, PL, and TLW, and generally favored higher values and showed consistent grouping (**Figure 4**).

**Table 6:** List of promising rice bean accessions identified as superior over the best check variety (bold) used in this study based on the mean values for each environment.

Season/location	Delhi, 2019	Delhi, 2020	Delhi, 2021	Delhi, 2022	Almora, 2020	Almora, 2021	Mean
Days to 50% flowering (DF)							
EC934379-sel*	50.1	52.0	50.0	54.0	80.0	83.0	61.5
EC934368-sel	52.1	51.0	52.0	55.0	75.0	81.0	61.0
EC18261-sel	50.1	48.0	53.0	55.0	91.0	90.0	64.5
IC351508-sel	55.1	55.0	55.0	64.0	52.0	55.0	56.0
**PRR-1**	55.1	62.3	62.9	69.2	77.0	75.8	67.1
Days to 80% maturity (DM)							
EC934379-sel	–	78.0	75.0	80.0	120.0	126.0	95.8
EC18261-sel	–	75.0	73.0	81.0	140.0	139.0	101.6
EC934368-sel	–	78.0	80.0	84.0	115.0	124.0	96.2
IC351508-sel	–	80.0	80.0	81.0	75.0	80.0	79.2
**PRR-1**	–	101.1	111.9	110.2	121.1	115.3	111.9
Stem diameter (cm) (SD)							
IC369664-sel	14.1	10.7	16.7	–	7.3	9.7	11.7
IC526465-sel	12.4	13.0	14.8	–	9.2	8.7	11.6
IC552995-sel	9.7	10.0	15.7	–	12.6	7.3	11.0
IC397763-sel	18.9	9.3	9.9	–	9.5	8.0	11.1
**PRR-1**	8.3	7.8	10.7	–	8.4	8.3	8.7
Pod Length (cm) (PL)							
IC545608-sel	11.0	11.0	11.0		7.1	6.2	9.3
IC129123-sel	9.1	8.0	9.1	7.9	8.7	8.7	8.6
IC521101-sel	11.5	8.2	8.4	9.4	8.8	6.2	8.8
IC422927-sel	8.7	9.3	11.4	9.3	7.1	6.2	8.7
**RBL-6**	8.1	7.4	8.1	8.2	7.2	6.0	7.5
Number of seeds/pod (NSP)							
IC545608-sel	11.3	10.0	11.3		6.0	5.0	8.7
IC521096-sel	11.4	7.0	8.7	8.7	7.0	6.0	8.1
IC521177-sel	12.2	7.0	9.3	8.7	5.0	6.0	8.0
IC552976-sel	10.0	10.0	10.0		6.0	6.0	8.4
**VRB-3**	7.9	6.8	7.2	7.1	6.7	5.3	6.8
100 seed weight (g) (SW)							
IC569084-sel	14.3	12.0	10.0	12.5	8.0	8.7	10.9
IC352853-sel	22.2	19.0	20.0	16.4	9.0	7.6	15.7
IC422853-sel	17.2	12.0	10.0	10.1	7.9	8.0	10.8
IC449234-sel	17.2	10.5	17.2	12.5	9.9	7.3	12.4
**PRR-2**	5.7	4.6	5.9	6.3	8.2	7.9	6.4

*”-sel” suffix denotes selection, indicating single plant selection made during the characterization experiments and core development to maintain homogeneity.

Based on the multi-season multi-location data promising accessions which are superior than the best check and rest of accessions for traits such as early flowering, large pod length, a high seed count per pod and bold seed size were identified (**Table 6**). The accession IC351508 has shown extra early flowering (55–64 days) across the locations and also found to have a determinate growth habit and synchronous in maturity (**Table 6**; **Supplementary Figure 12**). Another genotype, IC352944, has shown determinate growth habit, synchronous maturity, as well as high grain yield (**Supplementary Figure 12**).

## 4 Discussion

Ricebean is a promising pulse and fodder crop owing to its nutritionally rich grains and inherent tolerance to a range of biotic and abiotic stresses. Despite these advantages, its production has not been scaled up, primarily due to the persistence of wild or semi-domesticated traits and the limited availability of information on the extent of genetic variation within ricebean germplasm collections. To date, only a few studies, largely based on relatively small sets of accessions, have attempted to evaluate genetic variability for morpho-agronomic traits in ricebean (Pattanayak et al., 2018). Consequently, breeding efforts in ricebean have been minimal and have relied mainly on simple selection approaches. In this context, the present study was undertaken to characterize the genetic variability present in ricebean accessions conserved in the Indian National Genebank and to identify a representative core set that can be effectively utilized in ricebean improvement programmes.

The present study demonstrates that ricebean core collections established by CoreHunter, MSTRAT, PowerCore, and PCSS sampling strategies were able to retain substantial phenotypic variation across 12 morpho-agronomic traits in this study. Similar to earlier methods reported in other crops (Odong et al., 2013; Krishnan et al., 2014; Archak et al., 2016), each method selected a distinct set of accessions and varied in how well they preserved diversity-related statistics, highlighting the strong influence of algorithm choice on core composition. Analysis of mean genetic distances (E-NE, A-NE, and E-E) for the core sets showed high retention of overall diversity, indicating that the cores were effectively representative of the entire collection. Complete coverage of phenotypic classes in all cores also confirmed that no method caused the loss of defined trait categories. However, differences became more evident when examining sensitive measures of representativeness and distribution, such as MD%, VD%, CR%, and VR%, which are recognized as reliable indicators of core collection quality (Hu et al., 2000; Zhang et al., 2012). This variability underscores the importance of evaluating multiple parameters rather than relying on a single metric. Among the approaches tested, the E-NE criterion with 100% weightage (E-NE:100) in CoreHunter produced the most robust subset, excelling in diversity and representativeness metrics. This core set met recommended thresholds of CR% (>80%) and VR% (>100%), demonstrating comprehensive coverage of the base collection and its variability. These results align with previous studies highlighting Core Hunter’s advantage over M-strategy and PCA-based methods in various crops, especially when balancing diversity and representativeness is crucial (Thachuk et al., 2009; Odong et al., 2013).

The final ricebean core set (E-NE:100) included 251 accessions, representing 14.3% of the base collection. While slightly above the conventional 10% benchmark, previous research indicates that larger cores are beneficial for conserving diversity in complex, quantitatively inherited traits (Noirot et al., 1996). Brown (1989) and Bisht et al. (1998) also reported that retaining 10% of the base collection typically preserves 75-90% of genetic variation, supporting the appropriateness of the chosen core size. Considering ricebean’s underutilized status and the need to broaden its genetic base, a relatively larger, highly representative core is both justified and desirable. Variations in the proportional representation of states or countries in the CS, relative to the EC, were observed and can be attributed to the strategy followed in the core development i.e., maximizing allelic richness. Consequently, regions harbouring lower genetic diversity contributed fewer accessions to the CS, whereas more diverse regions were proportionally better represented (**Supplementary Table 1**). Further, the identified core set (CS), i.e., E-NE:100, when compared with the entire collection (EC) for various diversity parameters such as descriptor states and phenotypic classes of qualitative traits, summary statics parameters and interquartile range (IQR) of distribution of quantitative traits indicated that the sampling technique to constitute the core collection was appropriate. QQ plot provides a superior visual representation of the differences in distribution of a specific trait between the CS and the EC (Odong et al., 2013). The QQ plots for quantitative traits indicate that the distribution of the entire ricebean collection is optimally represented by the established core collection of 251 accessions. The analogous trend of the *H′* index in both CS and EC suggests that the ricebean core collection effectively represents the overall diversity of the collection. The *H’* index as a measure of core set validation has been widely employed by many researchers (Bisht et al., 1998; Upadhyaya et al., 2003; Upadhyaya et al., 2001, 2006; Mahalakshmi et al., 2007; Upadhyaya et al., 2009; Phogat et al., 2020).

A proper sampling is crucial for developing a representative core collection and therefore, must consider the phenotypic correlations, as crop evolves with co-adapted gene complexes (Ortiz et al., 1998). The established core set (CS) maintained magnitude and direction of relationships among traits identified in the entire collection (EC). The pattern of correlations observed in ricebean are also reported in earlier studies such as mungbean (Gayacharan et al., 2020), chickpea (Upadhyaya et al., 2001) and finger millet (Upadhyaya et al., 2010). Similarly, principal component analysis indicated almost same magnitude of variance explained by the first five PCs in both type of populations with a slightly higher values for CS. However, significantly higher variance was explained by PC1 in CS (35.05%) than EC (28.78%). The information on pattern and magnitude of vector loadings across the PCs can be useful in selection of traits for target breeding program.

An additional criterion for validating core collections involves analyzing the spatial distribution of accessions and elucidating variance through principal component analysis (Bisht et al., 1998; Wang et al., 2008). Although the trait’s grid mapping showed traits’ diversity relationship with the geographical location, hierarchical clustering based on the eight quantitative traits showed that trait variation has no significant relationship with the geographical origin of the ricebean collections.

Genotypes’ differential responses across locations, seasons or management conditions remain one of the major challenges in plant breeding, because it weakens the predictability of field performance, making selection and varietal recommendation more complex. Therefore, we have also studied the genotype × environment (G×E) interactions to identify genotype performance and stability across diverse environments as described by (Yan and Kang, 2002). A large proportion of the variance explained by the first two PCs for the critical phenological characteristics (Days to flowering, Days to maturity, and Seed weight) suggests these traits are highly affected by genotype and environment in a trait-specific G×E response pattern. Therefore, stable and early to medium duration accessions identified for DF and DM across environments could be useful in breeding short-duration ricebean varieties suitable for regions with limited flowering period, whereas late-maturing-high-biomass accessions are more suited to regions having longer post-monsoon vegetative phase that can act as donors in fodder-based breeding. The consistently discriminative and representative Delhi environment indicates its usefulness for general selection and mainstream breeding, while the highly discriminative but less representative Almora environment seems to be more suitable for assessing genotype sensitivity and specific adaptability. Developmental traits such as DF and DM, as well as sink-related yield components like SW, often exhibit higher heritability and lower genotype × environment interaction compared to other complex traits (Hallauer et al., 2010; Saltz et al., 2018). Studies indicate that this is because such traits are under stronger genetic control (Zhang et al., 2015; Saltz et al., 2018), which also results in higher heritability as revealed in this study (**Table 5**).

The trait-specific superior genotypes identified in this study can serve as donors in developing the ideal ricebean cultivars through breeding programs to overcome the major drawbacks such as indeterminate growth habit and asynchronous maturity, to bring the ricebean crop in mainstream cropping systems. Genotypes viz., IC351508 and IC352944, which have determinate growth habit and synchronous flowering, can be highly useful in ricebean improvement for developing cultivars suitable for mechanization and large-scale cultivation. Similarly, other trait specific promising accessions may serve as a valuable resource to expand the genetic diversity of ricebean breeding programs and the development of new cultivars. Further, the availability of ricebean core set (CS) may allow for the selective examination of similar clusters or geographical areas associated with trait specific accessions, thereby incorporating diversity into genetic improvement programs. Following similar approach, core collections of minor crops, including finger millet (Upadhyaya et al., 2010), barnyard millet (Sood et al., 2015) and foxtail millet (Sharma et al., 2014) were found to be very useful in the identification of trait specific accessions for yield contributing traits, quality and stress tolerance. Being ricebean intercrossable with other *Vigna* species (Arora et al., 1980; Pandiyan et al., 2008) and highly resistant for various biotic stresses, the findings of this study may find usefulness in the improvement of other Vigna group of crops.

## 5 Conclusion

This study presents the first comprehensive effort to assemble, evaluate, and validate a core collection (251 accessions) of ricebean germplasm representing the global diversity conserved in the Indian National Genebank. The rigorous comparison of sampling methods and subsequent statistical validation using diversity indices, variance retention, range representation, and correlation structure confirmed that the final core set preserves both the richness as well as representativeness of ricebean diversity. Multi-environment evaluations of the core set further revealed meaningful genotype × environment interactions, particularly for flowering time, maturity, seed weight, and pod traits. Importantly, the reduced size of the ricebean collections will enable large-scale multilocational testing, disease screening, and genomic characterization, thereby accelerating the discovery of valuable alleles for breeding. Because ricebean is cross-compatible with several *Vigna* species, the trait-rich accessions identified in this study also have the potential to contribute to the improvement of other underutilized pulses. Overall, the ricebean core collection established here has potential for enhancing the use of genetic resources in breeding programs. The study will be helpful in systematic trait discovery and the development of climate-resilient cultivars, and it strengthens the foundation for genomic-assisted breeding in ricebean and related *Vigna* crops.

## Data Availability

The original contributions presented in the study are included in the article/**Supplementary Material**. Further inquiries can be directed to the corresponding authors.
