# Correction: Multi-environment phenotyping of ricebean (*Vigna umbellata* (Thunb.) Ohwi & Ohashi) germplasm and identification of core set for accelerating the crop improvement programs

**DOI:** 10.3389/fpls.2026.1961548

**Published:** 2026-10-30

**Authors:** Gayacharan, Dinesh C Joshi, J Aravind, D P Wankhede, Badal Singh, Prakash Kumar, S Rajkumar, Swarup K Parida, D P Semwal, Paras Sharma, Mohar Singh, Debasis Chattopadhyay, Kuldeep Singh, G. P. Singh, Amit Kumar Singh

**Affiliations:** 1 ICAR-National Bureau of Plant Genetic Resources, New Delhi, India; 2 ICAR- Vivekananda Parvatiya Krishi Anusandhan Sansthan, Almora, India; 3 ICAR- Indian Agricultural Statistics Research Institute, New Delhi, India; 4 BRIC-National Institute of Plant Genome Research, New Delhi, India; 5 ICMR‐National Institute of Nutrition (NIN), Hyderabad, Telengana, India; 6 ICAR-National Bureau of Plant Genetic Resources, R S, Shimla, India; 7 International Crops Research Institute for the Semi-Arid Tropics, Patancheru, Hyderabad, India

**Keywords:** core development, core evaluation, core hunter, diversity analysis, potential crop

There was a mistake in **Table 6** as published. The term “-sel” has been added to the accession ID (in the first column).

**Table 6:** List of promising rice bean accessions identified as superior over the best check variety (bold) used in this study based on the mean values for each environment.

Season/location	Delhi, 2019	Delhi, 2020	Delhi, 2021	Delhi, 2022	Almora, 2020	Almora, 2021	Mean
Days to 50% flowering (DF)							
EC934379-sel*	50.1	52.0	50.0	54.0	80.0	83.0	61.5
EC934368-sel	52.1	51.0	52.0	55.0	75.0	81.0	61.0
EC18261-sel	50.1	48.0	53.0	55.0	91.0	90.0	64.5
IC351508-sel	55.1	55.0	55.0	64.0	52.0	55.0	56.0
**PRR-1**	55.1	62.3	62.9	69.2	77.0	75.8	67.1
Days to 80% maturity (DM)							
EC934379-sel	–	78.0	75.0	80.0	120.0	126.0	95.8
EC18261-sel	–	75.0	73.0	81.0	140.0	139.0	101.6
EC934368-sel	–	78.0	80.0	84.0	115.0	124.0	96.2
IC351508-sel	–	80.0	80.0	81.0	75.0	80.0	79.2
**PRR-1**	–	101.1	111.9	110.2	121.1	115.3	111.9
Stem diameter (cm) (SD)							
IC369664-sel	14.1	10.7	16.7	–	7.3	9.7	11.7
IC526465-sel	12.4	13.0	14.8	–	9.2	8.7	11.6
IC552995-sel	9.7	10.0	15.7	–	12.6	7.3	11.0
IC397763-sel	18.9	9.3	9.9	–	9.5	8.0	11.1
**PRR-1**	8.3	7.8	10.7	–	8.4	8.3	8.7
Pod Length (cm) (PL)							
IC545608-sel	11.0	11.0	11.0		7.1	6.2	9.3
IC129123-sel	9.1	8.0	9.1	7.9	8.7	8.7	8.6
IC521101-sel	11.5	8.2	8.4	9.4	8.8	6.2	8.8
IC422927-sel	8.7	9.3	11.4	9.3	7.1	6.2	8.7
**RBL-6**	8.1	7.4	8.1	8.2	7.2	6.0	7.5
Number of seeds/pod (NSP)							
IC545608-sel	11.3	10.0	11.3		6.0	5.0	8.7
IC521096-sel	11.4	7.0	8.7	8.7	7.0	6.0	8.1
IC521177-sel	12.2	7.0	9.3	8.7	5.0	6.0	8.0
IC552976-sel	10.0	10.0	10.0		6.0	6.0	8.4
**VRB-3**	7.9	6.8	7.2	7.1	6.7	5.3	6.8
100 seed weight (g) (SW)							
IC569084-sel	14.3	12.0	10.0	12.5	8.0	8.7	10.9
IC352853-sel	22.2	19.0	20.0	16.4	9.0	7.6	15.7
IC422853-sel	17.2	12.0	10.0	10.1	7.9	8.0	10.8
IC449234-sel	17.2	10.5	17.2	12.5	9.9	7.3	12.4
**PRR-2**	5.7	4.6	5.9	6.3	8.2	7.9	6.4

*”-sel” suffix denotes selection, indicating single plant selection made during the characterization experiments and core development to maintain homogeneity.

The corrected **Table 6** appears below.

The original version of this article has been updated.

