# Supplementary material for: Multi-environment phenotyping of ricebean (Vigna umbellata (Thunb.) Ohwi & Ohashi) germplasm and identification of core set for accelerating the crop improvement programs
Source: Front Plant Sci. 2026 Mar 20;17:1757675. doi: 10.3389/fpls.2026.1757675 (PMC13047085; doi:10.3389/fpls.2026.1757675)
Supplement: Supplementary file 1 [file DataSheet1.docx]

**Table S1:** Origin distribution of ricebean germplasm used in this study

| **Type** | **Country Source/origin** | **No. of accessions** | |
| --- | --- | --- | --- |
|  |  | **Entire collections** | **Core set** |
| EC | Australia | 1 | 0 |
| EC | Bolivia | 1 | 0 |
| EC | Belgium | 2 | 0 |
| EC | Brazil | 2 | 0 |
| EC | Cango | 1 | 1 |
| EC | China | 1 | 1 |
| EC | Guatemala | 1 | 0 |
| EC | Indonesia | 3 | 3 |
| EC | Japan | 11 | 11 |
| EC | Mexico | 1 | 0 |
| EC | Myanmar | 1 | 1 |
| EC | Nepal | 24 | 12 |
| EC | Papua New Guinea | 1 | 1 |
| EC | Russia | 3 | 0 |
| EC | Taiwan | 6 | 6 |
| EC | United States of America | 5 | 1 |
| EC | Unknown | 2 | 0 |
| IC | Andaman and Nicobar Islands | 1 | 1 |
| IC | Andhra Pradesh | 61 | 14 |
| IC | Arunachal Pradesh | 74 | 9 |
| IC | Assam | 10 | 3 |
| IC | Bihar | 7 | 2 |
| IC | Chhattisgarh | 3 | 2 |
| IC | Delhi | 3 | 2 |
| IC | Haryana | 11 | 4 |
| IC | Himachal Pradesh | 84 | 12 |
| IC | Jharkhand | 15 | 8 |
| IC | Karnataka | 1 | 1 |
| IC | Madhya Pradesh | 9 | 3 |
| IC | Manipur | 67 | 15 |
| IC | Meghalaya | 214 | 27 |
| IC | Mizoram | 30 | 3 |
| IC | Nagaland | 100 | 11 |
| IC | Odisha | 58 | 23 |
| IC | Punjab | 37 | 3 |
| IC | Sikkim | 86 | 13 |
| IC | Tamil Nadu | 1 | 0 |
| IC | Tripura | 2 | 1 |
| IC | Uttar Pradesh | 39 | 4 |
| IC | Uttarakhand | 64 | 15 |
| IC | West Bengal | 94 | 16 |
| IC | Unknown | 452 | 22 |
|  | **Total** | **1589** | **251** |

*IC=Indigenous collections (Indian); EC=exotic collections (introduced to India)

**Table S2:** Bartlet test for comparison of phenotypic quantitative data between two environments i.e., Delhi-2019 and Almora-2020

| Trait | **χ^2^ test value** | **d.f.** | **p-value** | **Significance** |
| --- | --- | --- | --- | --- |
| Days to 50% flowering | 131.13 | 1 | 2.3E-30 | ** |
| No. of branches/plant | 32.42 | 1 | 1.2E-08 | ** |
| Terminal leaf length (cm) | 0.06 | 1 | 8.1E-01 | ns |
| Terminal leaf width (cm) | 3.84 | 1 | 5.0E-02 | ns |
| Plant stem diameter (cm) | 454.73 | 1 | 6.7E-101 | ** |
| Pod length (cm) | 10.22 | 1 | 1.4E-03 | ** |
| No. of seeds/pod | 10.47 | 1 | 1.2E-03 | ** |
| 100 seed weight (g) | 1.16 | 1 | 2.8E-01 | ns |

**Table S3:** Basic passport information for ricebean core collection set (CS)

| **Sr. No.** | **Acc. ID** | **Date of Collection** | **Collector/Alternate ID** | **Cultivar Name** | **District** | **State** | **Country** |
| --- | --- | --- | --- | --- | --- | --- | --- |
| 1 | IC551628 | 03-15-07 | LRB-195 | Raj moong | Shillong | Meghalaya | India |
| 2 | EC550040 | 10-07-2004 | PI 208460, Masyang |  |  |  | Nepal |
| 3 | IC369664 | 02-16-02 | NDS-260 | Cuttiagulu | Vizianagaram | Andhra Pradesh | India |
| 4 | IC444194 | 10-18-04 | AKS/RSR-357 | Rajmoong | Pauri | Uttarakhand | India |
| 5 | IC521356 | 11-02-2004 | RKH-7 | Moth | Bilaspur | Himachal Pradesh | India |
| 6 | IC312394 | 03-04-2001 | VKG17/62 |  | East Singhbhum | Jharkhand | India |
| 7 | IC551651 | 03-15-07 | LRB-124 | Raj moong | Shillong | Meghalaya | India |
| 8 | EC18171 | 04-18-61 | Bobhamyia |  |  |  | Nepal |
| 9 | IC15663 | 06-19-70 | NIC No.-09 |  | Pakur | Jharkhand | India |
| 10 | IC552970 | 01-25-04 | KRB - 87 | Gaimung | Jalpaiguri | West Bengal | India |
| 11 | IC564858 | 08-02-2005 | KRB-153 | Gaimung | Murshidabad | West Bengal | India |
| 12 | IC26973 |  | NIC No-97 |  | Imphal West | Manipur | India |
| 13 | IC15664 | 06-19-72 |  |  | Pakur | Jharkhand | India |
| 14 | IC564714 | 01-19-08 | NSA/08/036 | Alasandalu | Visakhapatnam | Andhra Pradesh | India |
| 15 | IC137180 | 01-01-2000 | RB-39-B |  |  | Haryana | India |
| 16 | IC545608 | 01-05-2003 | KRB-65 | Gaimung | Purulia | West Bengal | India |
| 17 | EC615199 | 03-13-08 | Hong Kong HK2 |  |  |  | Taiwan |
| 18 | IC129089 |  | D-19 |  |  | Nagaland | India |
| 19 | IC417099 | 11-16-03 | UR-70 | Singji | Upper Siang | Arunachal Pradesh | India |
| 20 | IC524518 | 03-05-2005 | SS-AKM-164 |  | Dimapur | Nagaland | India |
| 21 | IC521096 | 03-23-05 | LRB-372 |  |  | Others | India |
| 22 | IC444138 | 10-13-04 | AKS/RSR-301 | Rajmoong | Pauri | Uttarakhand | India |
| 23 | IC433853 | 03-13-04 | DRLT-1732 | Moshum | Lohit | Arunachal Pradesh | India |
| 24 | IC342231 | 06-12-2000 | ORB-53 | Dangarrani | Koraput | Odisha | India |
| 25 | IC567233 | 11-14-08 | JAS/08-09 |  | M.Andaman | Andaman and Nicobar Islands | India |
| 26 | IC15668 | 06-19-72 |  |  | Pakur | Jharkhand | India |
| 27 | IC526465 | 02-07-2005 | SS-312 |  | Lunglei | Mizoram | India |
| 28 | EC550039 | 10-07-2004 | PI 200841 |  |  |  | Myanmar |
| 29 | EC934430 |  | JP No42099 |  |  |  | Japan |
| 30 | IC552995 | 02-06-2004 | KRB - 113 | Gaimung | Jalpaiguri | West Bengal | India |
| 31 | IC112383 |  |  |  | Shillong | Meghalaya | India |
| 32 | IC273820 | 04-27-00 | IGR-6-2000-6 | Bhadi | Bastar | Madhya Pradesh | India |
| 33 | IC520930 | 03-23-05 | LRB-141-1 |  |  | Others | India |
| 34 | IC137157 | 10-10-1985 | CXN-26 | Ghurush | Bhowali | Uttarakhand | India |
| 35 | IC392346 | 02-27-03 | AKP-10/27 | - | Sahibganj | Jharkhand | India |
| 36 | IC129120 |  | PRRS-2 |  |  | Delhi | India |
| 37 | EC18278 | 04-18-61 | Sorhedi |  |  |  | Nepal |
| 38 | IC137205 |  | RBL-70 |  |  | Punjab | India |
| 39 | IC342232 | 06-12-2000 | ORB-54 | Dangarrani | Koraput | Odisha | India |
| 40 | IC262754 | 04-30-00 | BDS/SG3050 | Mah/Mash | Mandi | Himachal Pradesh | India |
| 41 | IC129069 |  | CXM-11-P5-1 |  |  | Meghalaya | India |
| 42 | IC313497 | 03-01-2000 | SDS-4294 | Ricebean | Bastar | Madhya Pradesh | India |
| 43 | IC521177 | 03-23-05 | VUM-22 |  |  | Others | India |
| 44 | IC140810 |  | RBL-50 |  |  | Punjab | India |
| 45 | IC346026 | 05-05-2002 | TRS/RKC-1100 | Mari | Mandi | Himachal Pradesh | India |
| 46 | IC311947 | 12-16-00 | SS-156 | Kutting | Kandhanala | Odisha | India |
| 47 | IC564865 | 10-02-2005 | KRB-161 | Gaimung | Murshidabad | West Bengal | India |
| 48 | IC19351 | 03-31-73 | NIC NO.-100 |  | Phulbani | Bihar | India |
| 49 | EC615198 | 03-13-08 | Hong kong HK1 |  |  |  | Taiwan |
| 50 | IC343929 | 12-25-01 | SKN-120 | Minumulu | Visakhaptanam | Andhra Pradesh | India |
| 51 | IC352931 | 12-14-01 | BKSB-145 | Chakhawai | Thoubal | Manipur | India |
| 52 | IC342245 | 04-10-2000 | ORB-69 | Dangarrani | Rayagada | Odisha | India |
| 53 | IC521224 | 03-23-05 | BRS-1-A |  |  | Others | India |
| 54 | IC551662 | 03-15-07 | LRB-53 | Raj moong | Shillong | Meghalaya | India |
| 55 | IC116122 | 03-31-89 | N-278 |  | Nainital | Uttarakhand | India |
| 56 | IC397763 | 01-25-03 | HD-AKD/17 | Koli hawai khawew machu | Cachar | Assam | India |
| 57 | IC569120 | 11-01-2008 | M-006 | Rymbai-ja | West Khasi Hills | Meghalaya | India |
| 58 | IC573518 | 01-01-2009 | KRB-299 | KRB-299 | East Khasi Hills | Meghalaya | India |
| 59 | IC551626 | 03-15-07 | LRB-56 | Raj moong | Shillong | Meghalaya | India |
| 60 | EC615200 | 03-13-08 | Hong Kong HK3 |  |  |  | Taiwan |
| 61 | IC6204 | 11-27-58 |  |  |  | Tripura | India |
| 62 | IC557293 | 12-12-2007 | BK-07-15 | Mashiam dal | South Sikkim | Sikkim | India |
| 63 | IC520997 | 03-23-05 | LRB-230 |  |  | Others | India |
| 64 | EC934413 |  | JP No100302 |  |  |  | Japan |
| 65 | EC18771 | 05-30-61 | Birathati |  |  |  | Nepal |
| 66 | IC144685 |  | CXN-16-P-1-1 |  | Shillong | Meghalaya | India |
| 67 | IC342219 | 06-10-2000 | ORB-38 | Katenga | Kalahandi | Odisha | India |
| 68 | IC416978 | 11-19-03 | UR-128 | Situng Rondon | Upper Siang | Arunachal Pradesh | India |
| 69 | IC557286 | 12-11-2007 | BK-07-08 | Mashiam dal | East Sikkim | Sikkim | India |
| 70 | IC26966 | 01-22-77 |  |  | Tamenglong | Manipur | India |
| 71 | IC352944 | 12-16-01 | BKSB-149 | Chakwai achaouba | Imphal East | Manipur | India |
| 72 | EC18201 | 04-18-61 | Ramadi |  |  |  | Nepal |
| 73 | IC26960 | 01-22-77 |  |  | Chandel | Manipur | India |
| 74 | IC19336 | 03-26-73 |  |  | Phulbani | Odisha | India |
| 75 | IC384034 | 10-30-02 | SSK-166 | Thivirlu | East Godavari | Andhra Pradesh | India |
| 76 | IC342247 | 04-11-2000 | ORB-71 | Dangarrani | Rayagada | Odisha | India |
| 77 | IC15643 | 06-19-72 |  |  | Dumka | Jharkhand | India |
| 78 | IC137193 |  | RXS-27-P3 |  |  | Sikkim | India |
| 79 | EC97882 | 11-09-1971 | PI-247687 |  |  |  | Cango |
| 80 | IC353877 | 01-29-02 | SMAR-909 | Timerlu | Vishakhapatnam | Andhra Pradesh | India |
| 81 | EC18183 | 04-18-61 | Bhumri |  |  |  | Nepal |
| 82 | IC419810 | 12-10-2003 | VASHM-PC-3246 | Safed Mash | Pithoragarh | Uttarakhand | India |
| 83 | EC18567 | 05-30-61 | Birathati |  |  |  | Nepal |
| 84 | IC318871 | 05-31-01 | RK/DS-310 | Moongi | Mandi | Himachal Pradesh | India |
| 85 | IC369230 | 11-15-02 | Laldhusri | - | Giridih | Jharkhand | India |
| 86 | EC142567 |  | V. 4090 |  |  |  | Taiwan |
| 87 | IC373244 | 01-01-2003 | AS-133 | Baragudi | Rayagada | Odisha | India |
| 88 | IC342374 | 07-09-2002 | RB-12 | Orho | Wokha | Nagaland | India |
| 89 | IC521041 | 03-23-05 | LRB-287 |  |  | Others | India |
| 90 | IC342576 | 12-08-2002 | MK3UK1/075 | Belia Urda | Surguja | Chattisgarh | India |
| 91 | IC129123 |  | PBL-13 |  |  | Unknown | India |
| 92 | IC426793 | 06-12-2003 | BAR-182-1 | Judumulu | Srikakulam | Andhra Pradesh | India |
| 93 | IC569084 | 12-10-2008 | HNS/RS-124 | Simba | Gajapati | Odisha | India |
| 94 | IC58553 |  | A0375 |  | Darjeeling | West Bengal | India |
| 95 | EC18136 | 04-18-61 | Moshang |  |  |  | Nepal |
| 96 | IC557317 | 12-16-07 | BK-07-39 | Mashiam dal | East Sikkim | Sikkim | India |
| 97 | EC934379 |  | JP No25211 |  |  |  | Japan |
| 98 | EC18261 | 04-18-61 | Matikhana |  |  |  | Nepal |
| 99 | IC129080 |  | NKG-110(A) |  |  | Himachal Pradesh | India |
| 100 | IC362094 | 11-21-99 | BDS-2848 | Moong | Mandi | Himachal Pradesh | India |
| 101 | IC573517 | 01-01-2009 | KRB-207 | KRB-207 | Karbi Along | Assam | India |
| 102 | IC16802-A | 12-30-72 |  |  | Thoubal | Manipur | India |
| 103 | IC351596 | 12-21-01 | BKSB-183 | Moissum | East Sikkim | Sikkim | India |
| 104 | IC552976 | 01-29-04 | KRB - 93 | Gaimung | Jalpaiguri | West Bengal | India |
| 105 | EC934274 |  | JP No239866 |  |  |  | Japan |
| 106 | IC16771 | 12-30-72 |  |  | Churachandpur | Manipur | India |
| 107 | IC256929 |  | RM-I-080 |  | Cuttack | Odisha | India |
| 108 | IC599882 | 11-28-11 | RSR/SKS-25 | Masum | East Sikkim | Sikkim | India |
| 109 | IC342240 | 04-08-2000 | ORB-63 | Dangarrani | Koraput | Odisha | India |
| 110 | IC137146 | 10-14-85 | BD-30-B |  | Khasi Hills | Meghalaya | India |
| 111 | IC108862 |  |  |  | Mandi | Himachal Pradesh | India |
| 112 | IC137174 | 10-10-1985 | RB-4B | Ghurush | Bhowali | Uttarakhand | India |
| 113 | IC435838 | 09-04-2004 | DRLT-1961 | Andeyu | Upper Dibang Valley | Arunachal Pradesh | India |
| 114 | IC129062 | 12-31-89 | CXM-12P3-1 |  |  | Manipur | India |
| 115 | EC934368 |  | JP No251211 |  |  |  | Japan |
| 116 | IC521156 | 03-23-05 | LRB-510 |  |  | Others | India |
| 117 | IC620831 | 01-01-2000 | Palam Rajmung 1 (RBHP-43) |  |  | Unknown | India |
| 118 | IC16796 | 12-30-72 |  |  | SENAPATI | Manipur | India |
| 119 | IC551712 | 03-15-07 | Vum-158 | Raj moong | Shillong | Meghalaya | India |
| 120 | IC129119 |  | AAH/16 |  |  | Delhi | India |
| 121 | IC551667 | 03-15-07 | LRB-106 | Raj moong | Shillong | Meghalaya | India |
| 122 | IC137182 | 01-01-2000 | NIC No-95 |  |  | Haryana | India |
| 123 | IC618587 | 06-02-2016 |  | Jawahar Rice bean 2 (JRBJ 05-4) | Jabalpur | Madhya Pradesh | India |
| 124 | IC426778 | 04-12-2003 | BAR-055-2 | Pedda bobberlu | Srikakulam | Andhra Pradesh | India |
| 125 | IC266206 | 10-08-1999 | VRK-264 | Rains | Chamoli | Uttar Pradesh | India |
| 126 | IC361364 | 06-05-2002 | NDB-2571 | Bhotiya dal | Tehri | Uttarakhand | India |
| 127 | IC426789 | 06-12-2003 | BAR-143-1 | Judumulu | Srikakulam | Andhra Pradesh | India |
| 128 | IC146240 | 08-03-1995 |  |  |  | Unknown | India |
| 129 | IC129088 |  | H-248 |  |  | Nagaland | India |
| 130 | IC343827 | 12-22-01 | SKN-018 | - | Srikakulam | Andhra Pradesh | India |
| 131 | IC116118 | 03-31-89 | CXN-7-B |  | Nainital | Uttarakhand | India |
| 132 | IC554712 | 01-05-2006 | NRB-22 | Naga dal | Khonoma | Nagaland | India |
| 133 | IC361365 | 06-05-2002 | NDB-2572 | Bhotiya dal | Tehri | Uttarakhand | India |
| 134 | IC146261 | 08-03-1995 |  |  |  | Unknown | India |
| 135 | IC524451 | 03-05-2005 | SS-AKM-102 |  | Dimapur | Nagaland | India |
| 136 | IC342246 | 04-10-2000 | ORB-70 | Dangarrani | Rayagada | Odisha | India |
| 137 | IC521101 | 03-23-05 | LRB-379 |  |  | Others | India |
| 138 | IC521194 | 03-23-05 | VUM-27 |  |  | Others | India |
| 139 | IC551722 | 03-15-07 | Vum-197 | Raj moong | Shillong | Meghalaya | India |
| 140 | IC129078 |  | A-1350A |  |  | Sikkim | India |
| 141 | IC112381 |  |  |  | Shillong | Meghalaya | India |
| 142 | IC352853 | 12-09-2001 | BKSB-122 | Chakwi achouba | Imphal East | Manipur | India |
| 143 | IC564828 | 03-02-2005 | KRB-122 | Gaimung | Nadia | West Bengal | India |
| 144 | IC422927 | 01-23-04 | APRB -19 | Bate | Mamit | Mizoram | India |
| 145 | IC521113 | 03-23-05 | LRB-407 |  |  | Others | India |
| 146 | IC552967 | 01-24-04 | KRB - 84 | Gaimung | Jalpaiguri | West Bengal | India |
| 147 | IC351508 | 12-13-01 | BKSB-160 | Gala dal | Darjeeling | West Bengal | India |
| 148 | IC259993 | 01-08-2000 | PLP/D99-484 | Dangar rani | Koraput | Odisha | India |
| 149 | IC129092 |  | PI-29097-1 |  |  | Unknown | India |
| 150 | IC260001 | 01-08-2000 | PLP/D99-492 | Rice bean | Koraput | Odisha | India |
| 151 | IC521363 | 11-03-2004 | RKH-14 | Moth | Sirmour | Himachal Pradesh | India |
| 152 | IC521146 | 03-23-05 | LRB-466 |  |  | Others | India |
| 153 | IC19338 | 03-26-73 |  |  |  | Odisha | India |
| 154 | EC114075 | 08-07-1975 | do |  |  |  | Indonesia |
| 155 | IC144701 |  | RXS-4-P-3 |  | Shillong | Meghalaya | India |
| 156 | IC364047 | 10-14-02 | RPD-9 | Lal massam | Mungan | Sikkim | India |
| 157 | IC137138 |  | BD-1-A |  |  | Nagaland | India |
| 158 | IC137183 | 01-01-2000 | RB-45-B |  |  | Haryana | India |
| 159 | IC141074 |  | BD-139-D |  |  | Meghalaya | India |
| 160 | IC342226 | 06-12-2000 | ORB-46 | Dangarrani | Koraput | Odisha | India |
| 161 | IC350791 | 01-07-2002 | BKSB-221 | Tohja | Jaintia Hill | Meghalaya | India |
| 162 | IC342242 | 04-08-2000 | ORB-66 | Dangarrani | Rayagada | Odisha | India |
| 163 | IC569073 | 12-08-2008 | HNS/RS-113 | Simba | Kalahandi | Odisha | India |
| 164 | EC98452 | 12-10-1971 | Krasnosemiannyj |  |  |  | Indonesea |
| 165 | IC137155 | 10-10-1985 | CXN-7-A |  |  | Nagaland | India |
| 166 | IC343841 | 12-22-01 | SKN-032 | - | Srikakulam | Andhra Pradesh | India |
| 167 | IC552977 | 01-29-04 | KRB - 94 | Gaimung | Jalpaiguri | West Bengal | India |
| 168 | IC538983 | 10-22-05 | SKS-126 | Amuperung | Lower Subansiri | Arunachal Pradesh | India |
| 169 | IC373406 | 10-27-02 | NR/02-141 | - | S.Kanara | Karnataka | India |
| 170 | EC615195 | 03-13-08 |  |  |  |  | Taiwan |
| 171 | IC350315 | 12-21-01 | PARS-782 | Timmerlu | Vishakhapatnam | Andhra Pradesh | India |
| 172 | IC129038 | 12-31-89 | RXS-27-P3 |  | Shillong | Meghalaya | India |
| 173 | IC341977 | 03-01-2000 | PRR1 |  | Almora | Uttarakhand | India |
| 174 | IC2074 | 04-18-53 | IC-2074A |  | Darjeeling | West Bengal | India |
| 175 | IC342577 | 12-09-2002 | MK3UK1/102 | Tusro | Surguja | Chattisgarh | India |
| 176 | IC144722 |  | NKG-137 |  | Shillong | Meghalaya | India |
| 177 | IC557283 | 12-11-2007 | BK-07-05 | Mashiam dal | East Sikkim | Sikkim | India |
| 178 | IC137141 | 10-14-85 | BD-14-A |  | Khasi Hills | Meghalaya | India |
| 179 | IC435833 | 09-04-2004 | DRLT-1956 | Andeyu | Upper Dibang Valley | Arunachal Pradesh | India |
| 180 | IC564861 | 09-02-2005 | KRB-156 | Gaimung | Murshidabad | West Bengal | India |
| 181 | IC350127 | 01-19-02 | BKSB-248 | Pincha | Changlang | Arunachal Pradesh | India |
| 182 | EC130191 | 07-21-79 | Wonsary market, C. java |  |  |  | Indonesia |
| 183 | IC129115 |  | VT-72/844-A |  |  | Uttar Pradesh | India |
| 184 | IC422853 | 01-20-04 | APRB - 15 | Bete | Mamit | Mizoram | India |
| 185 | IC369607 | 02-16-02 | NDS-201 | Cuttingulu | Vizianagaram | Andhra Pradesh | India |
| 186 | IC351696 | 12-26-01 | BKSB-196 | Methobean | West Sikkim | Sikkim | India |
| 187 | IC426783 | 04-12-2003 | BAR-064 | Bobberlu | Srikakulam | Andhra Pradesh | India |
| 188 | IC311942 | 01-09-2000 | SS-151 | Kutting | Kalahandi | Odisha | India |
| 189 | EC18113 | 04-18-61 | Pokhra Market |  |  |  | Nepal |
| 190 | IC342229 | 06-12-2000 | ORB-49 | Dangarrani | Koraput | Odisha | India |
| 191 | IC26962 | 01-22-77 |  |  | Tamenglong | Manipur | India |
| 192 | IC137166 |  | PLM-893-B |  |  | Punjab | India |
| 193 | IC521057 | 03-23-05 | LRB-320 |  |  | Others | India |
| 194 | EC165986 | 12-22-84 | PI251948 |  |  |  | Usa |
| 195 | IC336485 | 12-21-01 | VKG-20/37 | Rice bean (Suthro) | Jamui | Bihar | India |
| 196 | IC144700 |  | CXN-7-6 |  | Shillong | Meghalaya | India |
| 197 | IC144695 |  | CXN-42-P-2-1 |  | Shillong | Meghalaya | India |
| 198 | IC469185 | 01-01-1997 | CXM8-1-1 | Hara moong | Shimla | Himachal Pradesh | India |
| 199 | IC326994 | 10-29-01 | SKY-1022 | Ganga-Jamuna | Solan | Himachal Pradesh | India |
| 200 | IC554719 | 02-08-2006 | NRB-29 | Naga dal | Wokha | Nagaland | India |
| 201 | IC350152 | 01-20-02 | BKSB-250 | - | Changlang | Arunachal Pradesh | India |
| 202 | IC469177 | 01-01-1997 | CMX-68P-1 | Hara moong | Shimla | Himachal Pradesh | India |
| 203 | IC369663 | 02-16-02 | NDS-259 | Cuttiagulu | Vizianagaram | Andhra Pradesh | India |
| 204 | IC129067 |  | CXM-3/001-1 |  |  | Manipur | India |
| 205 | IC623660 | 12-02-2016 | SEJ 154 | Dangar rani | Kalahandi | Odisha | India |
| 206 | IC16706 | 11-09-1972 |  |  | Ukhrul | Manipur | India |
| 207 | IC573525 | 01-01-2009 | KRB-248 | KRB-248 | Eest Kalai Hill | Meghalaya | India |
| 208 | EC18566 | 05-30-61 | Birathati |  |  |  | Nepal |
| 209 | IC137169 | 10-10-1985 | PRR-8801-A | Ghurush | Tehri | Uttarakhand | India |
| 210 | EC18184 | 04-18-61 | Bhumri |  |  |  | Nepal |
| 211 | IC423374 | 12-23-03 | APRB-9 | anukchama | Mokokchung | Nagaland | India |
| 212 | EC934417 |  | JP No31442 |  |  |  | Japan |
| 213 | IC621805 | 09-27-16 | KCB/RSR/PKM-13 | Dhuans | Dehradun | Uttarakhand | India |
| 214 | IC469186 | 01-01-1997 | CXM5-1 | Hara moong | Shimla | Himachal Pradesh | India |
| 215 | IC521180 | 03-23-05 | VUM-224 |  |  | Others | India |
| 216 | IC16799 | 11-09-1972 | NIC No.-98 |  | SENAPATI | Manipur | India |
| 217 | EC615201 | 03-13-08 |  |  |  |  | Taiwan |
| 218 | IC137184 | 01-01-2000 | RB-46 |  |  | Haryana | India |
| 219 | IC545612 | 01-05-2003 | KRB-69 | Gaimung | Purulia | West Bengal | India |
| 220 | IC116113 | 03-31-89 | ARB-83(114-D) |  | Nainital | Uttarakhand | India |
| 221 | IC146260 | 08-03-1995 |  |  |  | Unknown | India |
| 222 | IC116129 | 03-31-89 | PI 29097A |  | Nainital | Uttarakhand | India |
| 223 | IC15642 | 06-19-72 |  |  | Latehar | Jharkhand | India |
| 224 | EC934263 |  | JP No227284 |  |  |  | Japan |
| 225 | EC114123 | 08-25-75 | IQ207/25-8-75DS |  |  |  | Papua |
| 226 | EC16167 | 03-07-1960 | Var. yang-liu-tou |  |  |  | China |
| 227 | IC573515 | 08-01-2009 | KRB-286 | KRB-286 | Ribhoi | Meghalaya | India |
| 228 | IC417127 | 11-18-03 | UR-96 |  | Upper Siang | Arunachal Pradesh | India |
| 229 | EC934424 |  | JP No99485 |  |  |  | Japan |
| 230 | IC521119 | 03-23-05 | LRB-419 |  |  | Others | India |
| 231 | IC521106 | 03-23-05 | LRB-393 |  |  | Others | India |
| 232 | IC554735 | 02-13-06 | NRB-45 | Naga dal | Makokuttony | Nagaland | India |
| 233 | EC934365 |  | JP No223041 |  |  |  | Japan |
| 234 | IC394316 | 03-05-2003 | SMBR-240 | Dirtu Pega | Dhemaji | Assam | India |
| 235 | IC129037 | 12-31-89 | CXM-12-P2-4 |  |  | Manipur | India |
| 236 | IC444172 | 10-16-04 | AKS/RSR-335 | Rajmoong | Tehri | Uttarakhand | India |
| 237 | IC199549 |  | RXS-8P2-1-2 |  |  | Sikkim | India |
| 238 | EC934409 |  | JP No100298 |  |  |  | Japan |
| 239 | IC137140 | 10-14-85 | BD-2-B |  | Khasi Hills | Meghalaya | India |
| 240 | IC129108 |  | CXN-P12-3-3 |  |  | Unknown | India |
| 241 | IC351621 | 12-22-01 | BKSB-185 | Sumoisseum | East Sikkim | Sikkim | India |
| 242 | EC934343 |  | JP No223033 |  |  |  | Japan |
| 243 | IC342239 | 06-15-00 | ORB-62 | Dangarrani | Koraput | Odisha | India |
| 244 | IC551632 | 03-15-07 | LRB-70 | Raj moong | Shillong | Meghalaya | India |
| 245 | IC316123 | 12-11-2000 | VRS-OM-1912 | Muad | Bareilly | Uttar Pradesh | India |
| 246 | IC351534 | 12-15-01 | BKSB-166 | Thulibhatamas | Darjeeling | West Bengal | India |
| 247 | IC449234 | 12-05-2004 | KG/PB-10 | Maskalai | Maldah | West Bengal | India |
| 248 | IC129097 |  | RXS-58-P3-2 |  |  | Sikkim | India |
| 249 | IC551711 | 03-15-07 | Vum-85 | Raj moong | Shillong | Meghalaya | India |
| 250 | IC311934 | 12-16-00 | SS-143 | Kutting | Phussari | Odisha | India |
| 251 | IC75061 | 06-17-60 |  |  |  | Uttar Pradesh | India |

**Table S4:** Clustering of core set accessions into sub-groups based on Ward’s function and UPGMA method

| Sr. No. | Acc | Ward's method | UPGMA method |
| --- | --- | --- | --- |
| 1 | IC551628 | C1 | G1 |
| 2 | EC550040 | C2 | G2 |
| 3 | IC369664 | C3 | G2 |
| 4 | IC444194 | C4 | G3 |
| 5 | IC521356 | C4 | G3 |
| 6 | IC312394 | C2 | G2 |
| 7 | IC551651 | C4 | G3 |
| 8 | EC18171 | C1 | G2 |
| 9 | IC15663 | C2 | G2 |
| 10 | IC552970 | C2 | G2 |
| 11 | IC564858 | C2 | G2 |
| 12 | IC26973 | C5 | G2 |
| 13 | IC15664 | C1 | G2 |
| 14 | IC564714 | C3 | G2 |
| 15 | IC137180 | C5 | G2 |
| 16 | IC545608 | C1 | G1 |
| 17 | EC615199 | C5 | G2 |
| 18 | IC129089 | C1 | G2 |
| 19 | IC417099 | C5 | G2 |
| 20 | IC524518 | C1 | G4 |
| 21 | IC521096 | C1 | G1 |
| 22 | IC444138 | C5 | G2 |
| 23 | IC433853 | C5 | G2 |
| 24 | IC342231 | C2 | G2 |
| 25 | IC567233 | C2 | G2 |
| 26 | IC15668 | C5 | G2 |
| 27 | IC526465 | C1 | G2 |
| 28 | EC550039 | C1 | G2 |
| 29 | EC934430 | C4 | G3 |
| 30 | IC552995 | C2 | G2 |
| 31 | IC112383 | C2 | G2 |
| 32 | IC273820 | C2 | G2 |
| 33 | IC520930 | C5 | G2 |
| 34 | IC137157 | C2 | G2 |
| 35 | IC392346 | C5 | G2 |
| 36 | IC129120 | C2 | G2 |
| 37 | EC18278 | C5 | G2 |
| 38 | IC137205 | C5 | G2 |
| 39 | IC342232 | C2 | G2 |
| 40 | IC262754 | C5 | G2 |
| 41 | IC129069 | C4 | G3 |
| 42 | IC313497 | C3 | G2 |
| 43 | IC521177 | C1 | G1 |
| 44 | IC140810 | C2 | G2 |
| 45 | IC346026 | C4 | G2 |
| 46 | IC311947 | C5 | G2 |
| 47 | IC564865 | C2 | G2 |
| 48 | IC19351 | C5 | G2 |
| 49 | EC615198 | C5 | G2 |
| 50 | IC343929 | C2 | G2 |
| 51 | IC352931 | C2 | G2 |
| 52 | IC342245 | C5 | G2 |
| 53 | IC521224 | C5 | G2 |
| 54 | IC551662 | C5 | G2 |
| 55 | IC116122 | C2 | G2 |
| 56 | IC397763 | C3 | G2 |
| 57 | IC569120 | C2 | G2 |
| 58 | IC573518 | C2 | G2 |
| 59 | IC551626 | C5 | G2 |
| 60 | EC615200 | C5 | G2 |
| 61 | IC6204 | C5 | G2 |
| 62 | IC557293 | C3 | G2 |
| 63 | IC520997 | C5 | G2 |
| 64 | EC934413 | C5 | G3 |
| 65 | EC18771 | C1 | G2 |
| 66 | IC144685 | C1 | G2 |
| 67 | IC342219 | C5 | G2 |
| 68 | IC416978 | C5 | G2 |
| 69 | IC557286 | C3 | G2 |
| 70 | IC26966 | C5 | G2 |
| 71 | IC352944 | C2 | G2 |
| 72 | EC18201 | C5 | G2 |
| 73 | IC26960 | C1 | G1 |
| 74 | IC19336 | C5 | G2 |
| 75 | IC384034 | C5 | G2 |
| 76 | IC342247 | C5 | G2 |
| 77 | IC15643 | C5 | G3 |
| 78 | IC137193 | C2 | G2 |
| 79 | EC97882 | C2 | G2 |
| 80 | IC353877 | C5 | G2 |
| 81 | EC18183 | C5 | G2 |
| 82 | IC419810 | C4 | G3 |
| 83 | EC18567 | C3 | G2 |
| 84 | IC318871 | C5 | G2 |
| 85 | IC369230 | C5 | G2 |
| 86 | EC142567 | C2 | G2 |
| 87 | IC373244 | C5 | G2 |
| 88 | IC342374 | C5 | G2 |
| 89 | IC521041 | C5 | G2 |
| 90 | IC342576 | C5 | G2 |
| 91 | IC129123 | C2 | G2 |
| 92 | IC426793 | C2 | G2 |
| 93 | IC569084 | C3 | G2 |
| 94 | IC58553 | C5 | G2 |
| 95 | EC18136 | C5 | G2 |
| 96 | IC557317 | C3 | G2 |
| 97 | EC934379 | C5 | G3 |
| 98 | EC18261 | C5 | G2 |
| 99 | IC129080 | C5 | G2 |
| 100 | IC362094 | C4 | G3 |
| 101 | IC573517 | C5 | G2 |
| 102 | IC16802-A | C3 | G2 |
| 103 | IC351596 | C5 | G2 |
| 104 | IC552976 | C1 | G1 |
| 105 | EC934274 | C4 | G3 |
| 106 | IC16771 | C5 | G2 |
| 107 | IC256929 | C5 | G2 |
| 108 | IC599882 | C2 | G2 |
| 109 | IC342240 | C2 | G2 |
| 110 | IC137146 | C5 | G2 |
| 111 | IC108862 | C2 | G2 |
| 112 | IC137174 | C5 | G2 |
| 113 | IC435838 | C2 | G2 |
| 114 | IC129062 | C2 | G2 |
| 115 | EC934368 | C5 | G3 |
| 116 | IC521156 | C5 | G2 |
| 117 | IC620831 | C4 | G2 |
| 118 | IC16796 | C5 | G2 |
| 119 | IC551712 | C1 | G2 |
| 120 | IC129119 | C5 | G2 |
| 121 | IC551667 | C5 | G2 |
| 122 | IC137182 | C5 | G2 |
| 123 | IC618587 | C5 | G2 |
| 124 | IC426778 | C1 | G2 |
| 125 | IC266206 | C5 | G2 |
| 126 | IC361364 | C4 | G3 |
| 127 | IC426789 | C5 | G2 |
| 128 | IC146240 | C5 | G2 |
| 129 | IC129088 | C5 | G2 |
| 130 | IC343827 | C6 | G5 |
| 131 | IC116118 | C5 | G2 |
| 132 | IC554712 | C3 | G2 |
| 133 | IC361365 | C4 | G3 |
| 134 | IC146261 | C5 | G2 |
| 135 | IC524451 | C2 | G2 |
| 136 | IC342246 | C1 | G2 |
| 137 | IC521101 | C1 | G1 |
| 138 | IC521194 | C1 | G2 |
| 139 | IC551722 | C1 | G4 |
| 140 | IC129078 | C5 | G2 |
| 141 | IC112381 | C5 | G2 |
| 142 | IC352853 | C3 | G2 |
| 143 | IC564828 | C2 | G2 |
| 144 | IC422927 | C1 | G2 |
| 145 | IC521113 | C1 | G1 |
| 146 | IC552967 | C2 | G2 |
| 147 | IC351508 | C5 | G2 |
| 148 | IC259993 | C1 | G2 |
| 149 | IC129092 | C5 | G2 |
| 150 | IC260001 | C2 | G2 |
| 151 | IC521363 | C2 | G2 |
| 152 | IC521146 | C1 | G1 |
| 153 | IC19338 | C5 | G2 |
| 154 | EC114075 | C4 | G3 |
| 155 | IC144701 | C5 | G2 |
| 156 | IC364047 | C3 | G2 |
| 157 | IC137138 | C1 | G4 |
| 158 | IC137183 | C1 | G2 |
| 159 | IC141074 | C5 | G2 |
| 160 | IC342226 | C5 | G2 |
| 161 | IC350791 | C3 | G2 |
| 162 | IC342242 | C1 | G2 |
| 163 | IC569073 | C4 | G3 |
| 164 | EC98452 | C1 | G2 |
| 165 | IC137155 | C1 | G2 |
| 166 | IC343841 | C6 | G5 |
| 167 | IC552977 | C1 | G1 |
| 168 | IC538983 | C5 | G2 |
| 169 | IC373406 | C5 | G2 |
| 170 | EC615195 | C5 | G2 |
| 171 | IC350315 | C1 | G2 |
| 172 | IC129038 | C5 | G2 |
| 173 | IC341977 | C1 | G2 |
| 174 | IC2074 | C5 | G2 |
| 175 | IC342577 | C5 | G2 |
| 176 | IC144722 | C5 | G2 |
| 177 | IC557283 | C3 | G2 |
| 178 | IC137141 | C1 | G2 |
| 179 | IC435833 | C5 | G2 |
| 180 | IC564861 | C1 | G2 |
| 181 | IC350127 | C5 | G2 |
| 182 | EC130191 | C3 | G2 |
| 183 | IC129115 | C5 | G2 |
| 184 | IC422853 | C1 | G2 |
| 185 | IC369607 | C3 | G2 |
| 186 | IC351696 | C2 | G2 |
| 187 | IC426783 | C2 | G2 |
| 188 | IC311942 | C5 | G2 |
| 189 | EC18113 | C1 | G2 |
| 190 | IC342229 | C5 | G2 |
| 191 | IC26962 | C5 | G2 |
| 192 | IC137166 | C1 | G2 |
| 193 | IC521057 | C1 | G2 |
| 194 | EC165986 | C5 | G6 |
| 195 | IC336485 | C2 | G2 |
| 196 | IC144700 | C1 | G2 |
| 197 | IC144695 | C5 | G2 |
| 198 | IC469185 | C5 | G2 |
| 199 | IC326994 | C5 | G2 |
| 200 | IC554719 | C3 | G2 |
| 201 | IC350152 | C3 | G2 |
| 202 | IC469177 | C4 | G3 |
| 203 | IC369663 | C2 | G2 |
| 204 | IC129067 | C2 | G2 |
| 205 | IC623660 | C2 | G2 |
| 206 | IC16706 | C1 | G2 |
| 207 | IC573525 | C3 | G2 |
| 208 | EC18566 | C5 | G2 |
| 209 | IC137169 | C2 | G2 |
| 210 | EC18184 | C5 | G2 |
| 211 | IC423374 | C3 | G2 |
| 212 | EC934417 | C5 | G3 |
| 213 | IC621805 | C4 | G2 |
| 214 | IC469186 | C5 | G2 |
| 215 | IC521180 | C7 | G7 |
| 216 | IC16799 | C5 | G2 |
| 217 | EC615201 | C5 | G2 |
| 218 | IC137184 | C5 | G2 |
| 219 | IC545612 | C2 | G2 |
| 220 | IC116113 | C5 | G2 |
| 221 | IC146260 | C1 | G2 |
| 222 | IC116129 | C5 | G2 |
| 223 | IC15642 | C1 | G2 |
| 224 | EC934263 | C4 | G3 |
| 225 | EC114123 | C5 | G3 |
| 226 | EC16167 | C5 | G2 |
| 227 | IC573515 | C5 | G2 |
| 228 | IC417127 | C5 | G2 |
| 229 | EC934424 | C4 | G3 |
| 230 | IC521119 | C1 | G1 |
| 231 | IC521106 | C1 | G1 |
| 232 | IC554735 | C2 | G2 |
| 233 | EC934365 | C5 | G3 |
| 234 | IC394316 | C5 | G2 |
| 235 | IC129037 | C2 | G2 |
| 236 | IC444172 | C5 | G3 |
| 237 | IC199549 | C5 | G2 |
| 238 | EC934409 | C4 | G3 |
| 239 | IC137140 | C1 | G2 |
| 240 | IC129108 | C5 | G2 |
| 241 | IC351621 | C4 | G2 |
| 242 | EC934343 | C4 | G3 |
| 243 | IC342239 | C2 | G2 |
| 244 | IC551632 | C5 | G2 |
| 245 | IC316123 | C5 | G2 |
| 246 | IC351534 | C2 | G2 |
| 247 | IC449234 | C5 | G2 |
| 248 | IC129097 | C5 | G2 |
| 249 | IC551711 | C1 | G4 |
| 250 | IC311934 | C1 | G2 |
| 251 | IC75061 | C5 | G2 |

**Table S5:** Analysis of variance (ANOVA) for the phenotypic traits recorded during the season 2019 at the Delhi location

| **Source** | **Degrees of freedom** | **Days to 50% flowering** | **No. of branches/plant** | **Terminal leaf length (cm)** | **Terminal leaf width (cm)** | **Plant stem diameter (cm)** | **Pod length (cm)** | **No. of seeds/pod** | **100 seed weight (g)** |
| --- | --- | --- | --- | --- | --- | --- | --- | --- | --- |
| Block (ignoring Treatments) | 39 | 3293.49 ** | 3.86 ** | 18.62 ** | 11.40 ** | 59.10 ** | 10.50 ** | 8.66 ** | 25.03 ** |
| Treatment (eliminating Blocks) | 1588 | 155.49 ** | 0.37 ⁿˢ | 1.71 ⁿˢ | 1.05 ⁿˢ | 2.74 ⁿˢ | 0.93 ** | 1.04 * | 4.41 ** |
| Treatment: Check | 3 | 2.92 ⁿˢ | 0.47 ⁿˢ | 1.15 ⁿˢ | 0.19 ⁿˢ | 0.06 ⁿˢ | 1.51 ⁿˢ | 1.10 ⁿˢ | 1.99 ** |
| Treatment: Test and Test vs. Check | 1585 | 155.78 ** | 0.37 ⁿˢ | 1.72 ⁿˢ | 1.05 ⁿˢ | 2.74 ⁿˢ | 0.93 ** | 1.04 * | 4.42 ** |
| Residuals | 120 | 4.27 | 0.30 | 1.81 | 0.86 | 2.40 | 0.67 | 0.74 | 0.45 |

ⁿˢ P > 0.05; * P <= 0.05; ** P <= 0.01

**Table S6:** Descriptive statistics of the quantitative phenotypic traits recorded on the entire collections of the ricebean germplasm during the monsoon season 2019

| **Trait** | **Min** | **Max** | **Mean** | **Std.Error** | **Std.Deviation** | **Skewness** | **Kurtosis** |
| --- | --- | --- | --- | --- | --- | --- | --- |
| DF | 42.73 | 125.13 | 69.33 | 0.35 | 13.92 | 1.36 ** | 5.08 ** |
| NBP | 2.36 | 14.36 | 3.75 | 0.02 | 0.64 | 3.20 ** | 50.97 ** |
| TLL (cm) | 4.39 | 17.17 | 10.37 | 0.04 | 1.42 | 0.04 ⁿˢ | 3.84 ** |
| TLW (cm) | 2.62 | 11.64 | 7.35 | 0.03 | 1.12 | 0.19 ** | 3.50 ** |
| SD (cm) | 3.52 | 18.94 | 8.93 | 0.05 | 1.81 | 0.57 ** | 4.38 ** |
| PL (cm) | 3.92 | 12.82 | 8.35 | 0.03 | 1.02 | 0.32 ** | 3.99 ** |
| NSP | 3.18 | 12.18 | 7.64 | 0.03 | 1.10 | 0.30 ** | 4.38 ** |
| SW (g) | 1.33 | 22.59 | 6.00 | 0.06 | 2.25 | 1.97 ** | 10.42 ** |

ⁿˢ P > 0.05; * P <= 0.05; ** P <= 0.01

Abbreviations: days to 50% flowering (DF), number of branches per plant (NBP), terminal leaf length (TLL) (cm), terminal leaf width (TLW) (cm), Plant stem diameter (SD) (mm), pod length (PL) (cm), number of seeds per pod (NSP), and seed weight (SW) (g)

**Table S7:** Frequency distribution of descriptor states of qualitative traits in entire collections (2019) and core set

| **Traits** | **Descriptor states (frequency)** | |
| --- | --- | --- |
|  | **Entire collection (EC)** | **Core set (CS)** |
| HYP_CLR | Green (1.6), Green purple (31.3), Purple (59.1), Dark purple (8.0) | Green (2.4), Green purple (35.2), Purple (50.4), Dark purple (12.0) |
| SED_VIG | Poor (1.4), Intermediate (12.5), Vigorous (86.1) | Poor (4.8), Intermediate (19.2), Vigorous (76.0) |
| FLR_CLR | Yellow (99.81), Purple-yellow (0.06), Other (0.12) | Yellow (98.67), Purple-yellow (0.44), Other (0.89) |
| SED_CLR | Dark green (1.8), Light green (50.8), Green-yellow (20.3), Yellow (1.1), Maroon (5.0), Brown (1.8), Navy Blue (5.4), Mottled green (7.9), Mottled brown (5.4), Mottled grey (0.6) | Dark green (4.0), Light green (31.2), Green-yellow (12.0), Yellow (2.4), Maroon (9.6), Brown (2.4), Navy Blue (10.4), Mottled green (12.8), Mottled brown (14.4), Mottled grey (0.8) |
| GRT_HBT | Erect (2.5), Semi-erect (6.8), Spreading (90.7) | Erect (6.4), Semi-erect (12.8), Spreading (80.8) |
| PLT_HBT | Semi-determinate (5.5), Indeterminate (94.5) | Semi-determinate (6.4), Indeterminate (93.6) |
| FLR_ABL | Asynchronous (66.3), Synchronous (33.7) | Asynchronous (64.0), Synchronous (36.0) |
| LFLT_SHP | Narrow (0.2), Intermediate (45.7), Rounded (51.3), Other (2.8) | Narrow (0.8), Intermediate (56.8), Rounded (35.2), Other (7.2) |
| LFLT_SIZ | Small (6.8), Medium (80.9), Large (12.3) | Small (12.0), Medium (72.0), Large (16.0) |
| POD_CLR | Light brown (39.8), Brown (58.9), Dark Brown (1.3) | Light brown (44.0), Brown (54.4), Dark Brown (1.6) |

Abbreviations***:*** HYP_CLR, hypocotyl colour; SED_VIG, seedling vigour; FLR_CLR, flower colour; SED_CLR, seed colour; GRT_HBT, growth habit; PLT_HBT, plant habit; FLR_ABL, flowering behaviour; LFLT_SHP, leaflet shape; LFLT_SIZ, leaflet size; POD_CLR, pod colour during near maturity

**Table S8:** Principal component analysis of multi-environment data of core set accessions

|  | **Terminal leaf length** | | |  | **Terminal leaf width** | | |
| --- | --- | --- | --- | --- | --- | --- | --- |
|  | PC1 | PC2 | PC3 |  | PC1 | PC2 | PC3 |
| **Eigen value** | 1.39 | 1.09 | 1.04 |  | 1.49 | 1.13 | 1.05 |
| **Proportion of variance** | 0.23 | 0.18 | 0.17 |  | 0.25 | 0.19 | 0.17 |
| **Cumulative variance** | 0.23 | 0.41 | 0.59 |  | 0.25 | 0.44 | 0.61 |
| **Vector loadings** |  |  |  |  |  |  |  |
| Delhi_2019 | **-0.74** | **-0.55** | -0.14 |  | **-0.57** | **0.74** | -0.24 |
| Delhi_2020 | **-0.63** | **0.74** | 0.23 |  | **-0.78** | **-0.60** | 0.06 |
| Delhi_2021 | -0.22 | -0.24 | -0.07 |  | -0.19 | 0.25 | **0.45** |
| Delhi_2022 | -0.08 | -0.18 | 0.17 |  | -0.03 | 0.13 | -0.16 |
| Almora_2020 | -0.05 | 0.25 | **-0.94** |  | -0.06 | 0.14 | **0.81** |
| Almora_2021 | 0.03 | 0.00 | -0.02 |  | 0.17 | 0.05 | 0.22 |
|  | **Days to 50% flowering** | | |  | **Days to 80% maturity** | | |
|  | PC1 | PC2 | PC3 |  | PC1 | PC2 | PC3 |
| **Eigen value** | 3.25 | 1.67 | 0.42 |  | 2.53 | 1.31 | 0.45 |
| **Proportion of variance** | 0.54 | 0.28 | 0.07 |  | 0.51 | 0.26 | 0.09 |
| **Cumulative variance** | 0.54 | 0.82 | 0.89 |  | 0.51 | 0.77 | 0.86 |
| **Vector loadings** |  |  |  |  |  |  |  |
| Delhi_2019 | **-0.51** | -0.06 | 0.17 |  | - | - | - |
| Delhi_2020 | **-0.54** | -0.13 | **-0.80** |  | **-0.61** | -0.21 | **0.76** |
| Delhi_2021 | **-0.54** | 0.16 | 0.30 |  | **-0.64** | 0.33 | **-0.49** |
| Delhi_2022 | -0.38 | 0.31 | 0.39 |  | **-0.43** | 0.16 | -0.18 |
| Almora_2020 | -0.11 | **-0.80** | 0.25 |  | -0.17 | **-0.68** | -0.33 |
| Almora_2021 | -0.04 | **-0.46** | 0.15 |  | -0.07 | **-0.60** | -0.21 |
|  | **No. of seeds/pod** | | |  | **Pod length** | | |
|  | PC1 | PC2 | PC3 |  | PC1 | PC2 | PC3 |
| **Eigen value** | 1.49 | 1.04 | 0.99 |  | 1.77 | 1.09 | 0.94 |
| **Proportion of variance** | 0.25 | 0.17 | 0.17 |  | 0.30 | 0.18 | 0.16 |
| **Cumulative variance** | 0.25 | 0.42 | 0.59 |  | 0.30 | 0.48 | 0.63 |
| **Vector loadings** |  |  |  |  |  |  |  |
| Delhi_2019 | **0.76** | 0.09 | **-0.54** |  | **-0.71** | 0.15 | -0.28 |
| Delhi_2020 | 0.31 | -0.10 | 0.15 |  | -0.29 | -0.06 | 0.13 |
| Delhi_2021 | 0.37 | -0.01 | 0.02 |  | **-0.51** | 0.17 | -0.04 |
| Delhi_2022 | **0.44** | -0.15 | **0.80** |  | -0.33 | 0.05 | **0.76** |
| Almora_2020 | -0.03 | **-0.98** | -0.19 |  | -0.13 | **-0.89** | 0.21 |
| Almora_2021 | -0.01 | 0.03 | 0.06 |  | -0.19 | -0.39 | **-0.53** |
|  | **Stem diameter** | | |  | **100 seed weight** | | |
|  | PC1 | PC2 | PC3 |  | PC1 | PC2 | PC3 |
| **Eigen value** | 1.51 | 1.09 | 0.98 |  | 2.74 | 1.06 | 0.88 |
| **Proportion of variance** | 0.30 | 0.22 | 0.20 |  | 0.46 | 0.18 | 0.15 |
| **Cumulative variance** | 0.30 | 0.52 | 0.72 |  | 0.46 | 0.63 | 0.78 |
| **Vector loadings** |  |  |  |  |  |  |  |
| Delhi_2019 | **0.68** | **0.67** | -0.30 |  | **-0.75** | **-0.42** | **0.50** |
| Delhi_2020 | 0.29 | 0.12 | **0.94** |  | **-0.46** | 0.29 | -0.33 |
| Delhi_2021 | **0.67** | **-0.73** | -0.11 |  | **-0.43** | 0.04 | **-0.69** |
| Delhi_2022 | - | - | - |  | -0.19 | **0.85** | **0.40** |
| Almora_2020 | -0.01 | -0.04 | 0.06 |  | -0.04 | 0.09 | -0.07 |
| Almora_2021 | -0.02 | 0.08 | 0.13 |  | 0.02 | 0.02 | -0.02 |


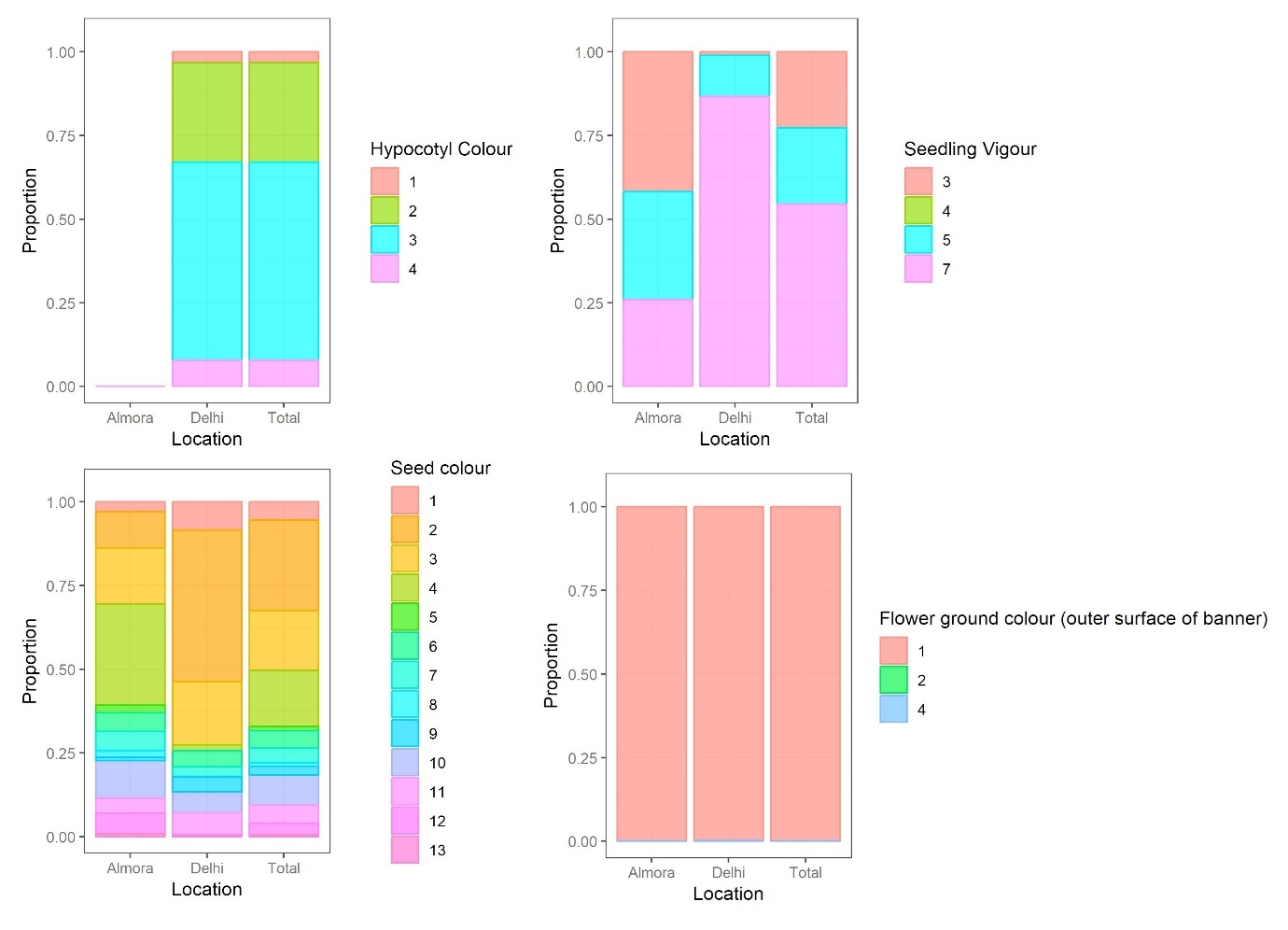


**Figure S1:** Bar chart frequency distribution of qualitative phenotypic parameters recorded on entire collections (EC) of ricebean at Delhi (2019) and Almora (2020) locations


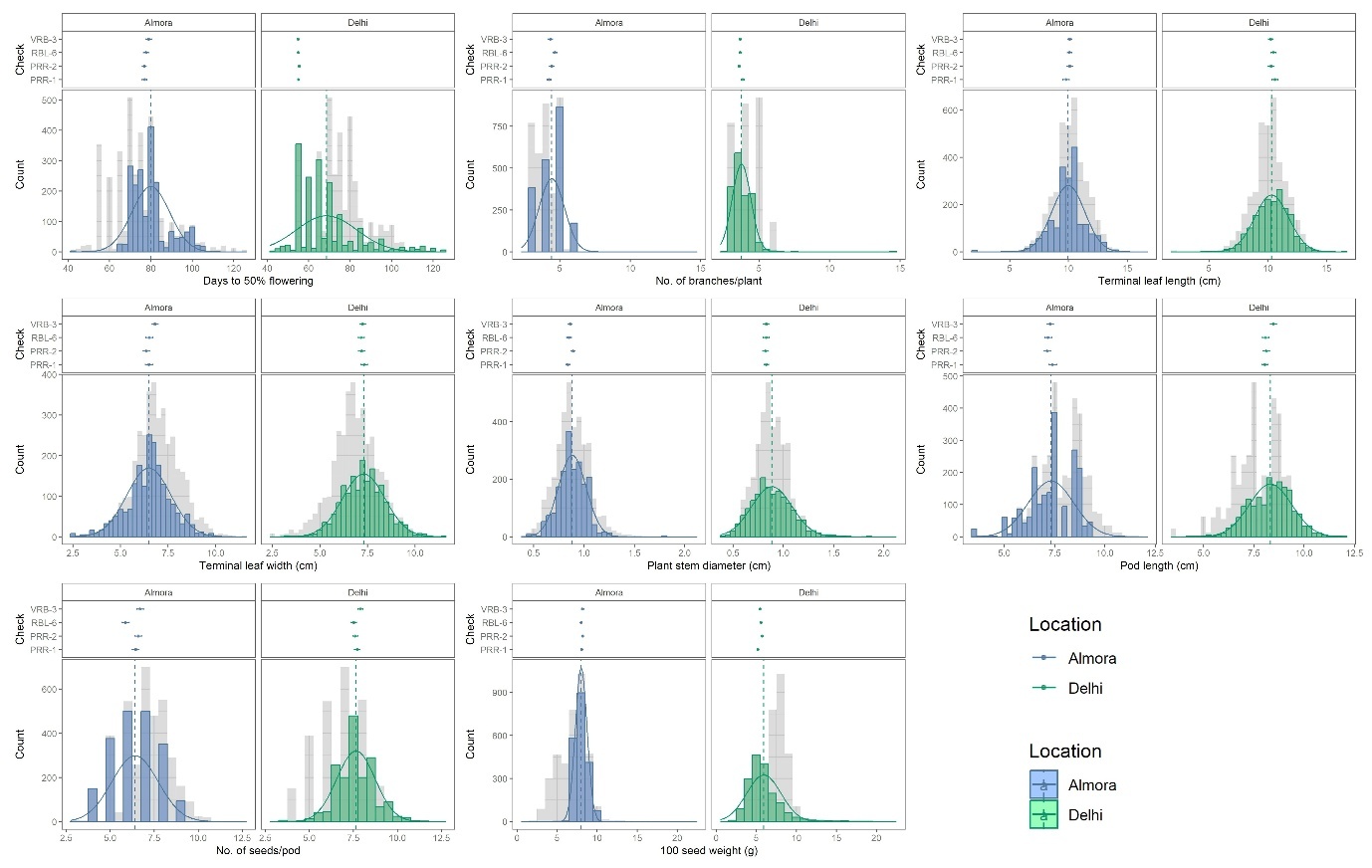


**Figure S2:** Bar chart frequency distribution of quantitative phenotypic traits recorded on entire collections (EC) of ricebean at Delhi (2019) and Almora (2020) locations


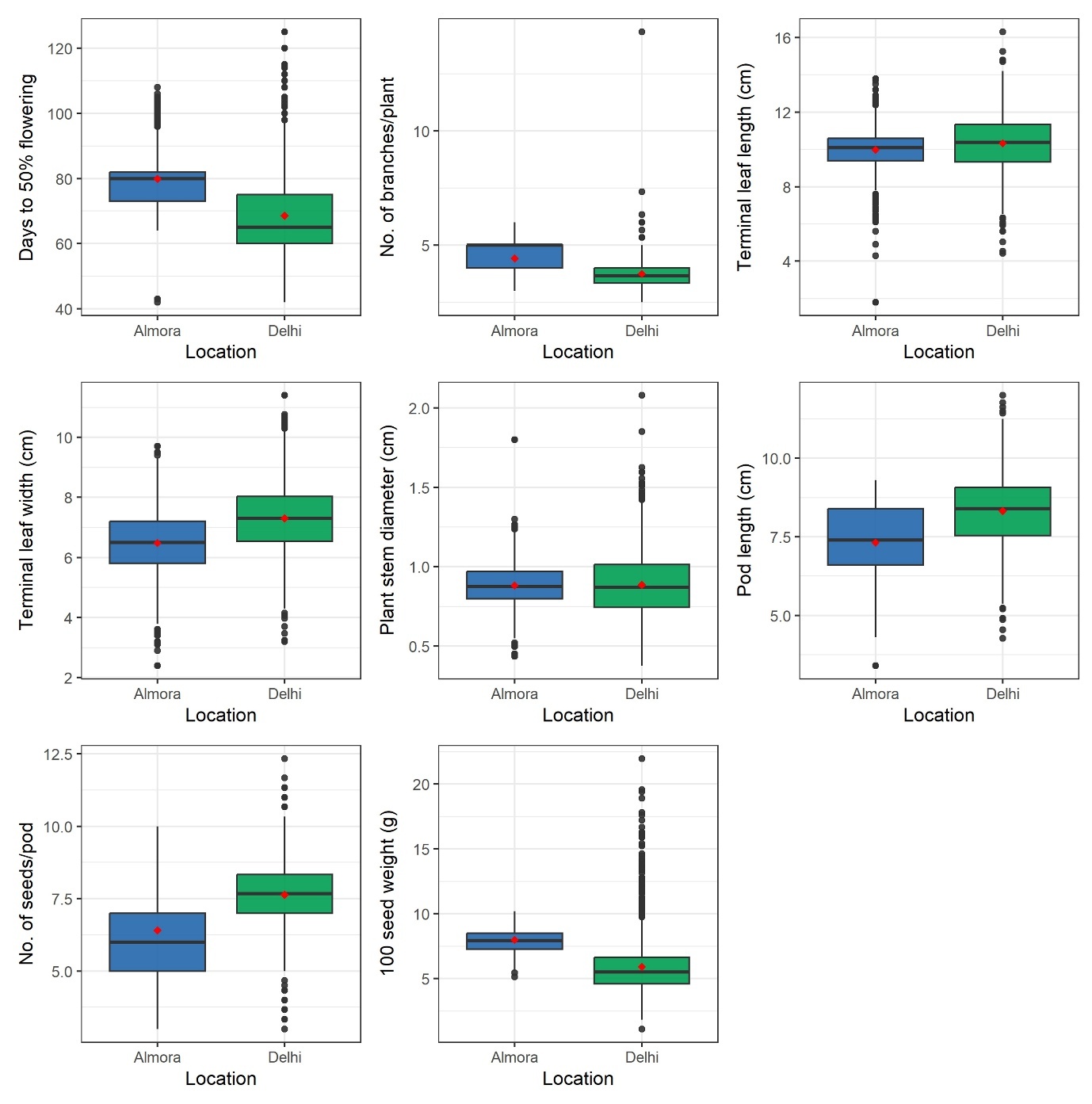


**Figure S3:** Box-plot frequency distribution of quantitative phenotypic traits recorded on entire collections (EC) of ricebean at Delhi (2019) and Almora (2020) locations


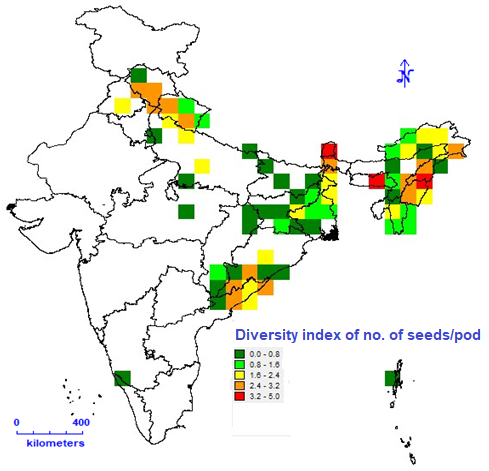


**Figure S4:** Grid map of India representing the diversity index for the number of seeds/pod (NSP) of ricebean germplasm


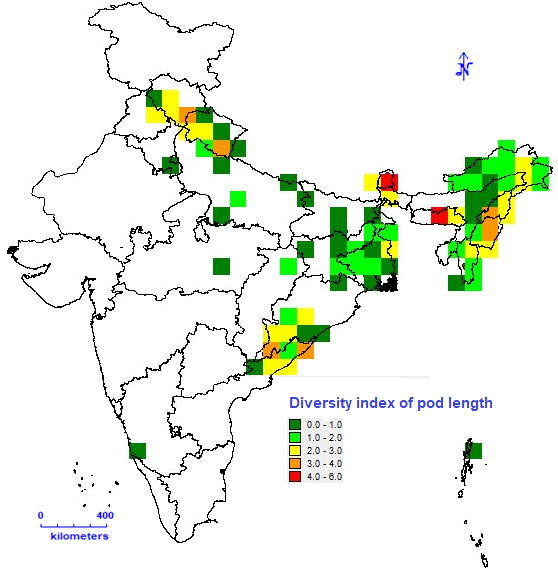


**Figure S5:** Grid map of India representing the diversity index for the number of pod length (PL) for ricebean germplasm


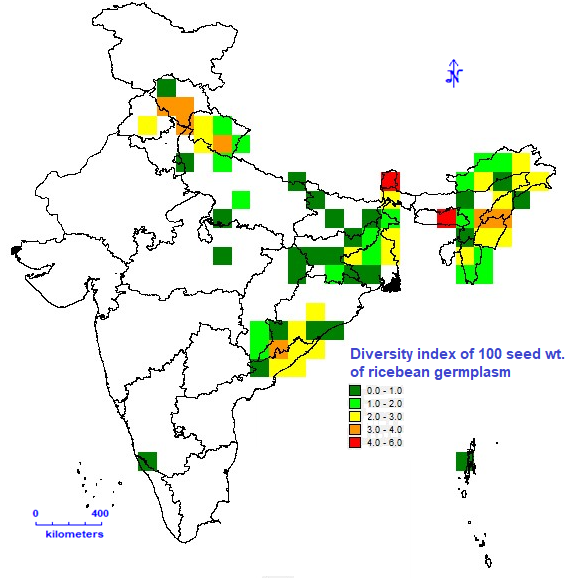


**Figure S6:** Grid map of India representing the diversity index for the 100 seed weight (SW) of ricebean germplasm


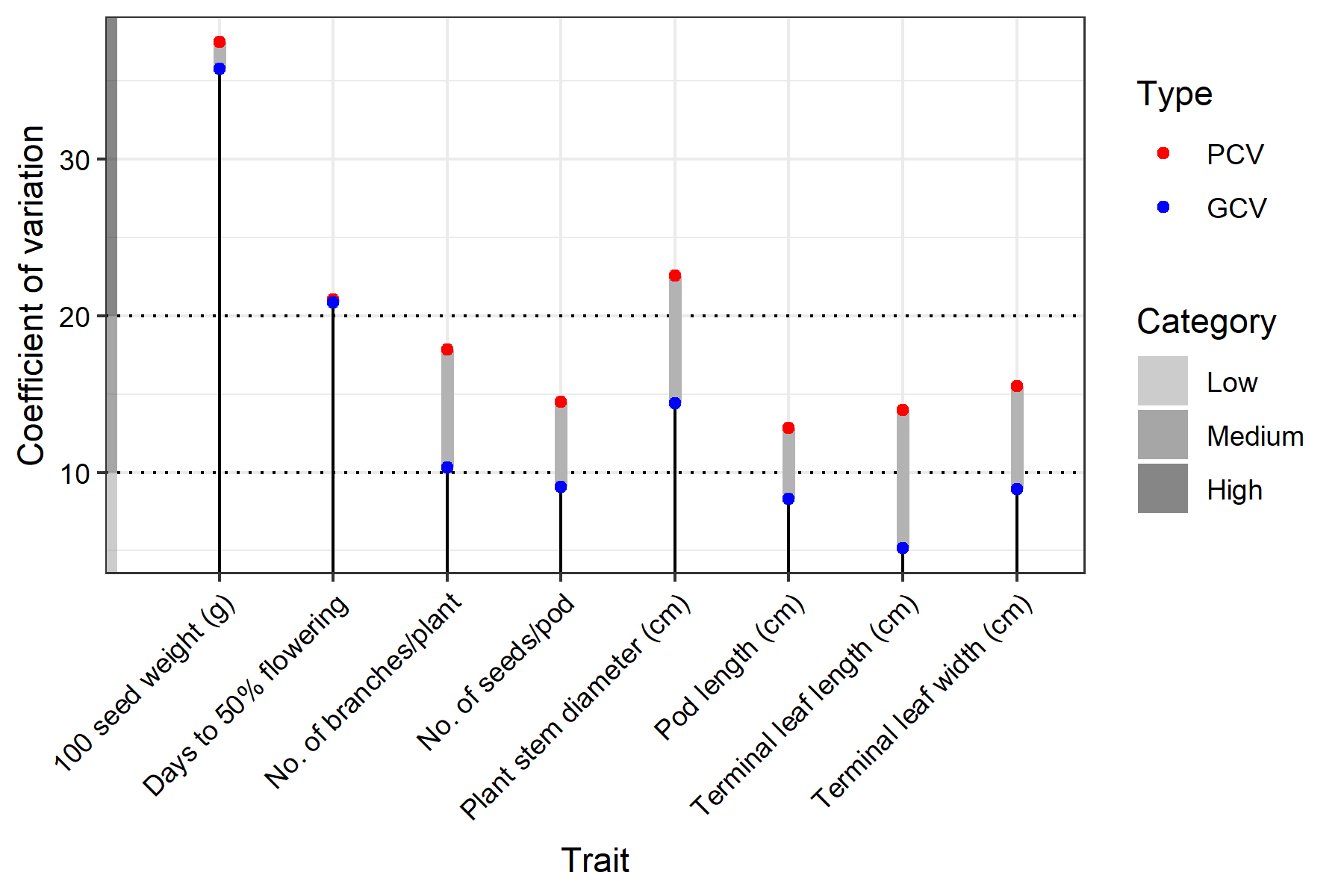


**Figure S7:** Gross value-added plot highlighting the GCV and PCV for quantitative phenotypic traits in the entire collection (EC) of mungbean germplasm


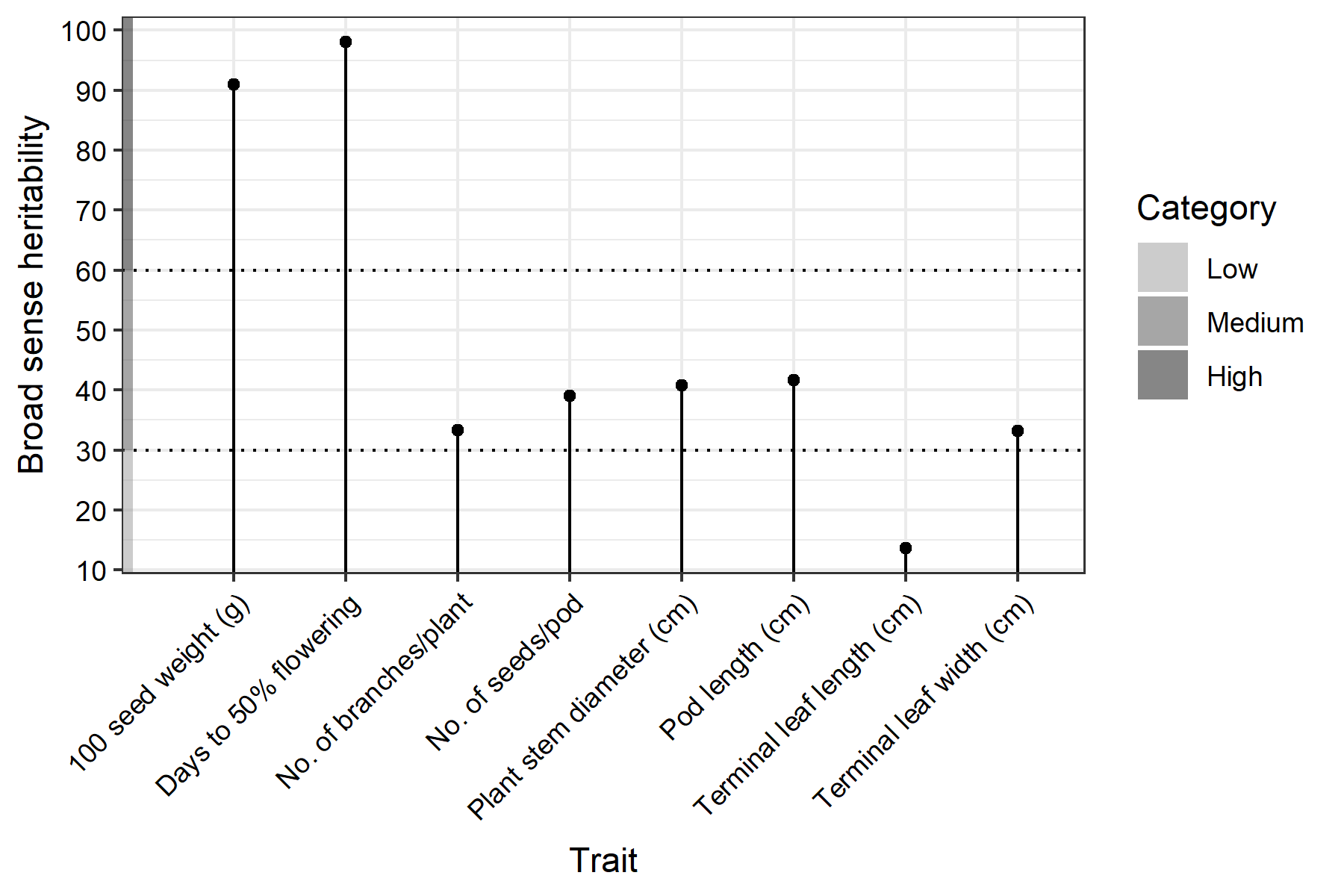


**Figure S8:** Gross value-added plot highlighting the broad-sense heritability (h^2^) for quantitative phenotypic traits in the entire collection (EC) of mungbean germplasm


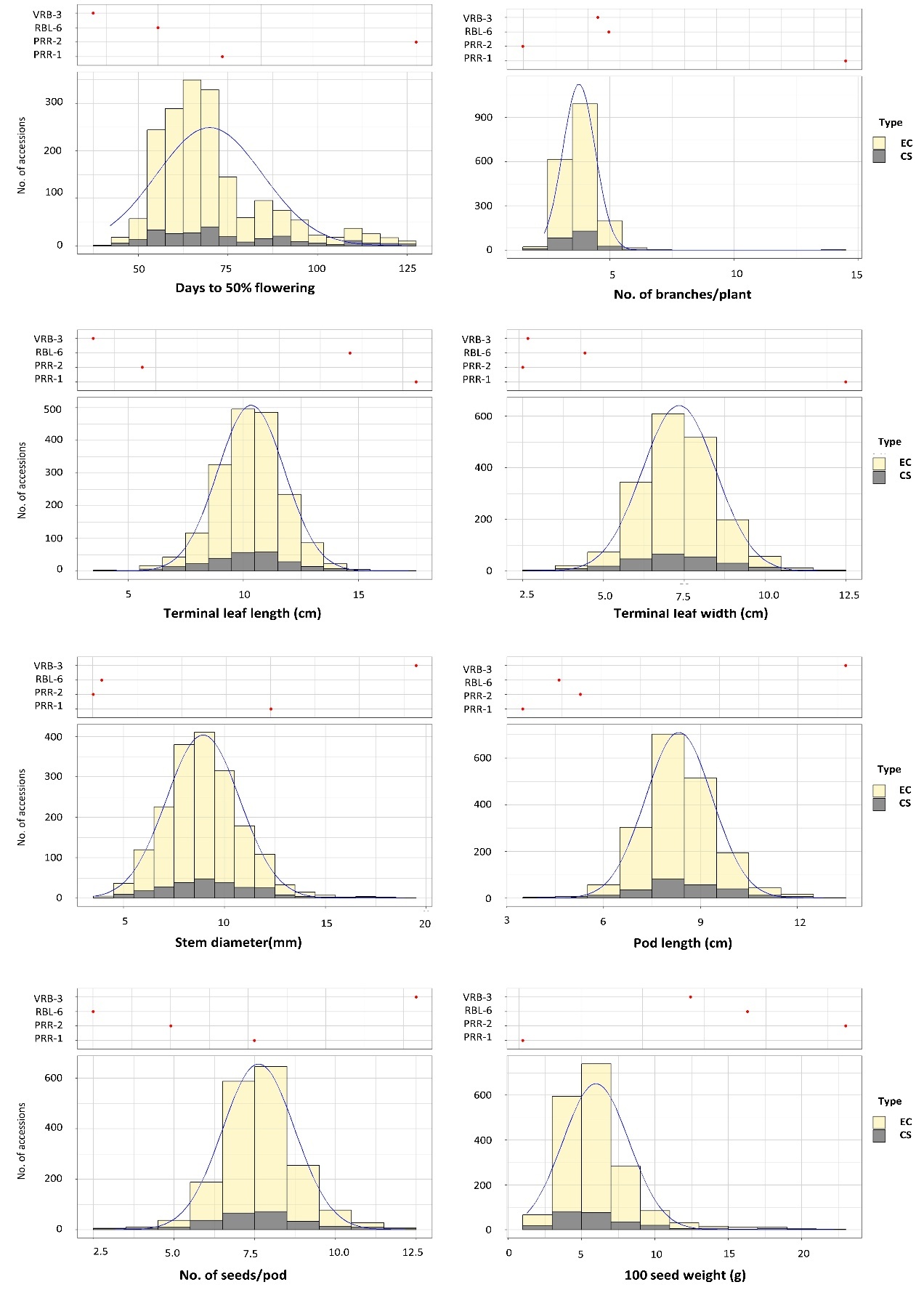


**Figure S9:** Frequency distribution chart of quantitative phenotypic parameters recorded on the entire collections (EC) and identified core set (CS) of ricebean. The frequency distribution indicates the representativeness of the diversity distribution for all the traits in the identified core set (CS).


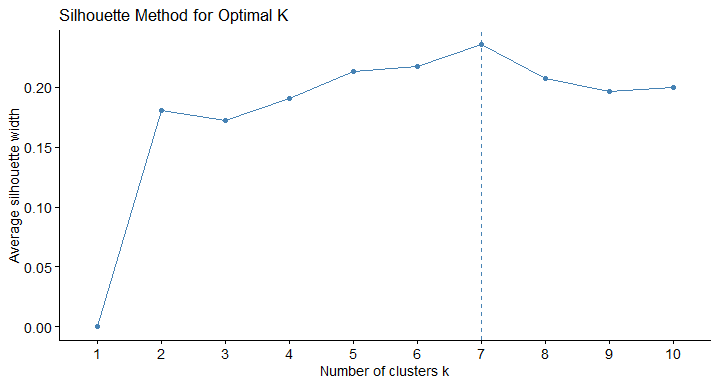


**Figure S10:** Determining the optimum number of clusters (*k*) based on the silhouette method in a core collection dataset by measuring how similar an accession is to its own cluster (cohesion) compared to other clusters (separation)


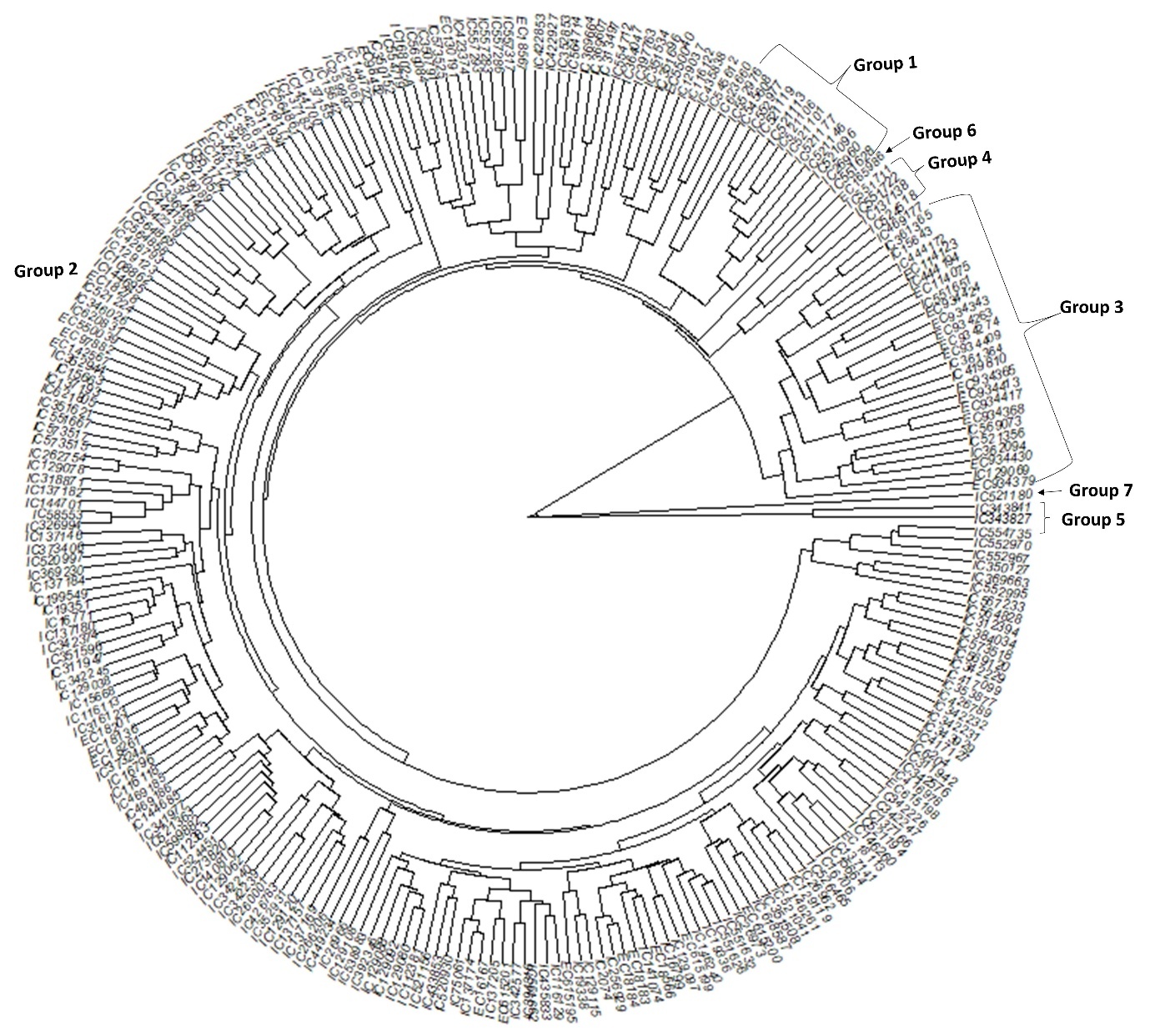


**Figure S11:** Fan layout of hierarchical clustering using the Unweighted Pair Group Method with Arithmetic Mean (UPGMA) based on average genetic distances among accessions of the designated core set (CS)


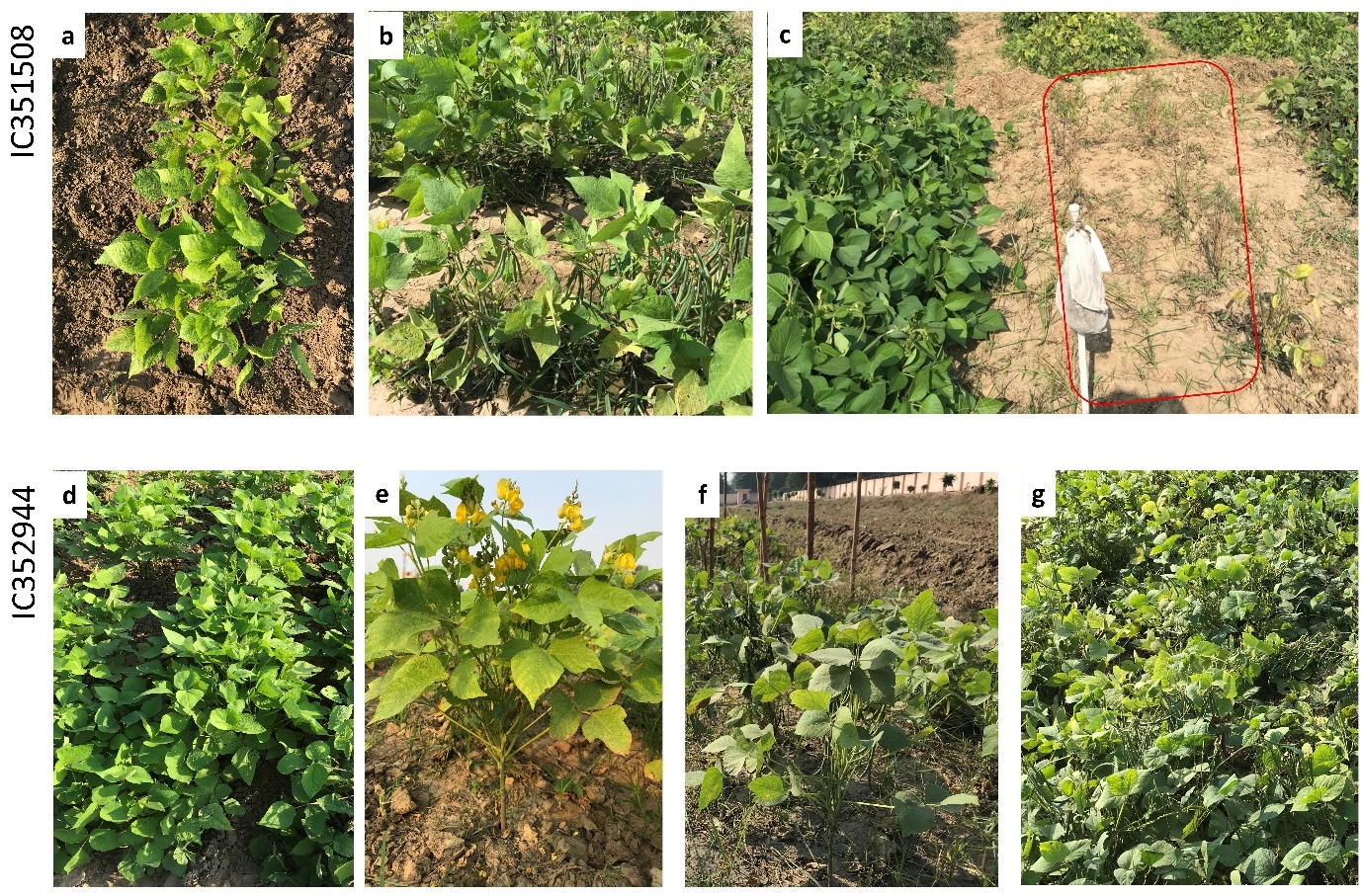


**Figure S12:** The genotypes IC351508 (a-c) and IC352944 (d-g) were identified to show a determinate growth habit during the evaluation of ricebean germplasm over multiple years (2019-2022) and locations (Delhi and Almora). The trait expression was consistent across the multiple seasons for both genotypes. This figure highlights the early vegetative growth (a) Podding stage (b), as well as early maturity within 75-80 days (c) of the ricebean genotype IC351508, and the similar vegetative growth (d), flowering habit (e), and shoot determinacy (f) were observed for the genotype IC352944. The early plant vigour and grain yield of the genotype IC35294 were also found to be relatively better, making it a promising genotype for ricebean breeding programs.
